# *Ligusticum chuanxiong* Hort as a medicinal and edible plant foods: Antioxidant, anti-aging and neuroprotective properties in *Caenorhabditis elegans*

**DOI:** 10.3389/fphar.2022.1049890

**Published:** 2022-10-26

**Authors:** Yihan Qin, Fangfang Chen, Zizhong Tang, Hongjiao Ren, Qing Wang, Nayu Shen, Wenjie Lin, Yirong Xiao, Ming Yuan, Hui Chen, Tongliang Bu, Qingfeng Li, Lin Huang

**Affiliations:** ^1^ College of Life Sciences, Sichuan Agricultural University, Ya’an, China; ^2^ Sichuan Agricultural University Hospital, Sichuan Agricultural University, Ya’an, China; ^3^ Triticeae Research Institute, Sichuan Agricultural University, Wenjiang, China

**Keywords:** Ligusticum chuanxiong, antioxidant activity, antiaging activity, neuroprotective activity, Caenorhabditis elegans

## Abstract

*Ligusticum chuanxiong* Hort. (CX) is a medicinal and edible plant including a variety of active substances, which may be an available resource for the treatment of related diseases. To expand the medicinal uses of CX, this study aims to explore the antioxidant, anti-aging and neuroprotective effects of the *Ligusticum chuanxiong* leaves (CXL) and rhizome (CXR) extracts. We first characterize CX phytochemical spectrum by LC-MS as well as antioxidant capacity. Acute toxicity, anti-oxidative stress capacity, lifespan and healthspan was evaluated in *C elegans* N2. Neuroprotective effect was evaluated *in vitro* and *in vivo* (*C elegans* CL4176 and CL2355). In this study, we detected 74 and 78 compounds from CXR and CXL, respectively, including phthalides, alkaloids, organic acids, terpenes, polyphenols and others. Furthermore, we found that CXs not only protect against oxidative stress, but also prolong the lifespan, alleviate lipofuscin, malondialdehyde (MDA) and reactive oxygen species (ROS) accumulation, and improve movement level, antioxidant enzyme activity in *C elegans* N_2_. However, only CXR reduced the *β*-amyloid peptide (Aβ)-induced paralysis phenotype in CL4176s and alleviated chemosensory behavior dysfunction in CL2355s. In addition, CXR treatment reduced the production of Aβ and ROS, enhanced SOD activity in CL4176s. The possible mechanism of anti-aging of CXL and CXR is to promote the expression of related antioxidant pathway genes, increase the activity of antioxidant enzymes, and reduce the accumulation of ROS, which is dependent on DAF-16 and HSF-1 (only in CXR). CXR was able to activate antioxidase-related (*sod-3* and *sod-5)* and heat shock protein genes (*hsp-16.1* and *hsp-70)* expression, consequently ameliorating proteotoxicity related to Aβ aggregation. In summary, these findings demonstrate the antioxidant, anti-aging and neuroprotective (only in CXR) activities of the CX, which provide an important pharmacological basis for developing functional foods and drugs to relieve the symptoms of aging and AD. However, the material basis of neuroprotective activity and antiaging effects need to be elucidated, and the relationship between these activities should also be clarified in future studies.

## 1 Introduction

Excessive ROS, a byproduct generated in an organism, could further induce oxidative stress and cause genomic stability together with damage to lipids and proteins, which has been linked to a number of chronic health problems, including aging, diabetes and neurodegenerative diseases et al. (Pisoschi and Pop, 2015; Aldosari et al., 2018; Lin et al., 2018). Aging is an inevitable process characterized by accumulating functional declines of physiological integrity that lead to impaired function and ultimately result in death (Fuellen et al., 2019). However, the number of older people is increasing rapidly worldwide, and aging has been recognized as a risk factor for age-related diseases, such as neurodegenerative diseases and metabolic diseases (Kaeberlein et al., 2015). Moreover, some neurodegenerative diseases, such as Alzheimer’s disease and Parkinson’s disease, seriously affect millions of people worldwide. So far, the medications to completely cure these diseases are unavailable or ineffective (Dimitriadi and Hart, 2010). However, increasing evidence suggests that many plants with highly abundant natural compounds which are consumed as antioxidant-rich foods or medicine to slow cellular senescence or aging, extend lifespan and treat age-related neurodegenerative diseases (Singh et al., 2019, 2021; Yadav et al., 2019). Therefore, there is great interest and urgency in the study of biological activity associated with health benefits in medicinal plants.

*Ligusticum chuanxiong* Hort (CX) belonging to the Umbelliferae, a traditional medicinal and edible plant, is commonly cultivated in Sichuan province in China (Chen et al., 2018). Traditionally, it is believed that the rhizome of CX is one of the most important and commonly used drugs for the therapy of gynecological diseases and hemiplegia (Chen et al., 2018). Moreover, the CX also can be consumed as a food, such as its tender leave has been used as tossed salad or fried cuisines and rhizome has been used for making tea, soup, and wine (Chen et al., 2018; Yuan et al., 2020). At present, the non-medicinal parts (leaves) of CX are favored by people all over the world. For example, in May 2021, Sichuan, China, for the first time exported 50 kg of fresh CX leaves to South Korea as a vegetable for sale in the South Korean market. Therefore, it is important to comprehensively investigate the health benefits and biological activity of its different parts. However, Previous research has largely focused on the chemical composition and pharmacological activity of CX’s rhizomes (Zhang et al., 2012; Li et al., 2014), ignoring the potential value of the leaves, which is not conducive to the comprehensive development and utilization of CX resources. Although studies have reported rhizomes of CX have rich phytochemicals with neuroprotective (Ling, 2018) and anti-aging (Jie et al., 2021) potential the leaves of CX possess the best antioxidant activity (Yan et al., 2022), scarce literatures are available regarding the underlying molecular mechanisms of CX extract extending lifespan. Therefore, more detailed studies are needed to support these claims.

There are more than 18,000 genes in the genome of *Caenorhabditis elegans* (*C*. *elegans*), of which 60% are homologues to human genes (Seauence and Biology, 1998). Moreover, besides the highly conserved metabolic pathways and similar physiological processes for humans, its easy-to-handling, short lifecycle and lifespan, and large mutant strains are some of the main advantages of the *C. elegans* (Qian et al., 2015). Therefore, the *C elegans* is a powerful tool to study the biological activity of natural products, allowing researchers the chance to preliminary study the potential benefits of plant extract, especially for testing their antioxidant and anti-aging capacities and their influence on some neurotoxic disorders (Yang et al., 2017).

Considering all the above, most of the previous research is focused on the active components and medicinal value of the medicinal parts of CX rhizome (Chen et al., 2018), and less attention has been given to the non-medicinal parts (leaves), which may cause a serious waste of resources. Therefore, we used *C. elegans* as a model to evaluate the potential biological activities and molecular mechanism of different parts of CX extract on neuroprotective, antioxidant and anti-aging effects. Our present investigation may promote the comprehensive utilization of CX as the potential dietary supplement or drug to delay the development of related diseases and promote health.

## 2 Materials and methods

### 2.1 Plant material and extraction

#### 2.1.1 Plant material and extraction

*Ligusticum chuanxiong* leaves (CXL) and rhizomes (CXR) were collected from the Sichuan Agricultural University farm in Ya’an City, Sichuan Province, China. The dried leaf and rhizome of *Ligusticum chuanxiong* were crushed into powder and subsequently extracted with absolute ethyl alcohol by soxhlet for 4 h at a temperature of 80–85°C. Then, by rotatory evaporation at 35–45°C and vacuum freeze drying, the solvent was removed. Finally, obtained CXL and CXR extract were dissolved in dimethyl sulfoxide (DMSO) to a final concentration of 100 mg/mL as stock solution.

### 2.2 *C. elegans* strains and maintenance conditions

*C. elegans* strains used: N2 (wild-type), CL4176 {dvIs2 [pCL12 (unc-54/human Aβ1-42 minigene) lpRF4}, CL2355 {dvIs50 [pCL45 (snb-1::Abeta1-42::3′UTR (long) + mtl-2::GFP)]I}, CL2122 {dvIs15[(pPD30.38) unc-54 (vector) + (pCL26) mtl-2::GFP]}, CF1038 [daf-16 (mu86) I], EU1 [skn-1 (zu67) IV/nT1 (IV; V)], PS3551 [hsf-1 (sy441) I]. All nematodes and *Escherichia coli* (OP50-uracil auxotorph) used in the study were obtained from the *Caenorhabditis* Genetics Center (CGC, University of Minnesota, Minneapolis, MN, United States). All strains were cultured at 20°C except the hypersensitive strains CL4176 and CL2355, which were cultured at 16°C. All strains were grown on nematode growth medium (NGM) and inactivated *E. coli* OP50 was the food source. All age-synchronized and contamination-free worms were obtained by an alkali-bleaching method except CL4176, CL2122, and CL2155, which were obtained by egg laying.

Unless otherwise specified, the treatment was as follows: 100 mg/ml storage solution and inactivated *E. coli* OP50 were mixed to prepare a treatment solution with a final concentration of 500 μg/ml, and then added to NGM. DMSO (0.5%) and Resveratrol (Res, 22.5 μg/ml) were used as a negative control (CK) and a positive control, respectively. Synchronized L1 larvae were then transferred onto the prepared NGM.

### 2.3 Analysis of plant chemical composition

#### 2.3.1 Determination of total phenolic and flavonoid contents

The total phenolic content was determined based on the method of Fan et al. (2019). A volume of 1.0 ml of Folin–Ciocalteu phenolic reagent was added 1.0 ml of two sample solutions (1 mg/ml), 5.0 ml of distilled water, and 3 ml of Na_2_CO_3_ (15%). The mixture was incubated for 2 h in the dark, then the absorbance was measured at 765 nm. Gallic acid was used as a standard for the calibration curve. The total phenolic content was expressed as mg of gallic acid equivalent/g of dry extract (GAE/mg of plant extracts).

The determination of total flavonoids was performed according to the aluminum chloride colorimetric method (Kim et al., 2003). 1 ml of the extract (1 mg/ml) mixed with distilled water (4 ml) in a test tube. Then, 0.3 ml of NaNO_2_ (5%) was added, followed by 0.3 ml of AlCl_3_ (10%). Test tubes were incubated at room temperature for 5 min, and then 2 ml of NaOH (1 M) was added to it. Immediately, the volume of the reaction mixture was made to 10 ml with distilled water and absorbance was measured at 510 nm.

#### 2.3.2 Liquid chromatography-mass spectrometry analysis

The 50 mg of CXL and CXR were each dissolved in 80% methanol. The mixture was sonicated at 4°C for 30 min, vortexed for 30 s, and finally centrifuged at 12,000 rpm for 15 min at 4°C. In brief, 5 µl of internal standard (0.14 mg/ml DL-o-chlorophenylalanine) was added to 200 µl of supernatant for analysis on UPLC-HRMS (Waters, UPLC; Thermo, Q Exactive) equipped with an ACQUITY UPLC BEH C18 column (2.1 × 100 mm i. d) with bead size of 1.7 µm. Chromatographic separation conditions are as follows: column temperature: 40°C; flow rate: 0.3 ml min^−1^ and injection volume: 0.5 µl. Gradient eluent is composed of 0.05% formic acid in water (A) and acetonitrile (B), and the gradient elution procedure was used: 0–1 min, 5% B; 2–13 min, 5%–95% B; 13–13.5 min; 95% B; 13.5–16 min, 95% B. For MS detection, ionization was performed in ESI+ and ESI- modes. The compounds were identified by comparing the retention times, mass spectra, and peak spiking with those found in literature and databases, such as MassBank (http://www.massbank.jp/), Phenol-Explorer (www.phenol-explorer.eu), Human Metabolome Database (https://hmdb.ca/), and PubChem (https://pubchem.ncbi.nlm.nih.gov).

### 2.4 Assessment of antioxidant activity *in vitro*

The antioxidant properties of CXR and CXL are investigated through *in vitro* chemical methods. Ascorbic acid (VC) was used as the positive control.

#### 2.4.1 ABTS radical scavenging assays

The ABTS free radical scavenging activity of samples were evaluated using a method described by Zhao et al., 2005. The 150 µl of sample solutions were mixed with 50 µl of ABTS solution for 6 min in a dark room. At 734 nm, the absorbance of the reaction mixture was measured (A_ABTS_). The scavenging activity of the ABTS radicals was calculated as follows:

$$Scavenging\;rate\;\left(\%\right)=\left[1-\left(A_{ABTS}-A_{Sample}\right)/A_{ABTS}\right]\times 100\%$$

#### 2.4.2 DPPH radical scavenging assays

DPPH radical scavenging assay followed the method described by Nuerxiati et al., 2019. In a 96-well plate, 100 μl of different dilutions of two extracts and 100 μl of DPPH ethanol solution (0.2 mM) were mixed and then incubated for 30 min at room temperature in the dark. The absorbance of the mixture was measured at 517 nm (A_DPPH_). Radical scavenging activity was expressed by the following equation:

$$Scavenging\;rate\;\left(\%\right)=\left[\left(A_{DPPH}-A_{Sample}\right)/A_{DPPH}\right]\times 100\%$$

#### 2.4.3 Ferric cyanide reducing power assay previously

The ferric-reducing property of CXL and CXR was determined using the method as described by Wang et al. (2012). The 2.5 ml tested samples with varying concentrations was mixed with 2.5 ml of phosphate buffer (0.2 M, pH 6.6) and 2.5 ml of 1% K_3_ [Fe(CN)_6_], which was cultured at 50°C for 30 min and cooled at room temperature. Subsequently, 2.5 ml of 10% trichloroacetic acid to mixture was added. Later, 5 ml of the supernatant collected by centrifugation at 5,000 rpm for 5 min and mixed with 4 ml of distilled water and 1 ml of 1.0% FeCl_3_. After 10 min of incubation, the absorbance of mixture was measured at 700 nm.

### 2.5 Acute toxicity assay

We performed the toxicity tests in accordance with Cristina’s description with some modifications (Moliner et al., 2018). The L4-stage synchronized populations were washed off twice with M9 buffer. Afterward, M9 buffer was mixed in a well with extracts and M9 as a negative control. In each treatment, 60 worms were exposed to extracts ranging from 25–1,000 μg/ml. The survival rate was measured at 20°C after 24 h.

### 2.6 Stress tolerance assay

L1-stage synchronized worms were transferred to medium containing CX extract (CXL or CXR; 500 μg/ml) until the first day of adulthood (approximately 3–5 days). They were then exposed to different stress conditions until all the worms died. For each condition, at least 120 worms were evaluated per condition. Dead worms were defined as ones that did not respond to a gentle touch with a platinum wire.

#### 2.6.1 H_2_O_2_-induced oxidative stress assay

The worms were transferred to freshly prepared NGM containing 2 mM H_2_O_2_ (Saul et al., 2008). The dead and live worms were examined every 30 min.

#### 2.6.2 Paraquat-induced oxidative stress assay

The worms were transferred to freshly prepared NGM containing 10 mM paraquat and was cultivated at 20°C (Lin et al., 2019). The vitality was examined every 24 h.

#### 2.6.3 Heat shock assay

In the thermotolerance assay, the worms were transferred to 35°C and the survival rate was recorded every hour (Lin et al., 2020).

### 2.7 Accumulations of intracellular reactive oxygen species and MDA, and activities of antioxidant enzymes

The CXL or CXR pretreated worms were collected and the total protein was extracted by ultrasonic treatment. Malondialdehyde (MDA), superoxide dismutase (SOD) and glutathione peroxidase (GSH-Px) activity was determined using the commercial assay kits (Nanjing Jiancheng Biotechnology Institute, China). Using three parallel samples, the test was conducted in triplicate.

ROS accumulation was measured with 2,7-dichlorodihydrofluorescein diacetate (H_2_DCF-DA) method (Büchter et al., 2013). Briefly, pretreated worms were collected and transferred to 96-well plates with the addition of 100 μl of 50 μM H_2_DCF-DA. Using an EnSpire multimode plate reader (PerkinElmer), the fluorescence intensity was read every 15 min for 6 h under the conditions of excitation at 485 nm, emission at 530 nm.

### 2.8 Lifespan assay

Based on a previously established method, the lifespan assay was conducted (Chen et al., 2014). L4-stage synchronized worms were transferred to NGM containing *E. coli* OP50 and CXL or CXR. The survival of worms was recorded daily. The experiment was performed three times with a total of 240 individuals.

### 2.9 Healthspan assays

#### 2.9.1 Movement assay

The movement assay was evaluated by three indicators: head swing, body bending and behavioral scoring. On the 3rd, 7th, and 11th day, worms were transferred to NGM. Then, the head swing times within 1 min and body bending times within 30 s of about 20 worms per group were counted under the microscope. Moreover, worms were observed and scored for classes A, B, and C using a stereomicroscope (Herndon et al., 2002).

#### 2.9.2 Lipofuscin accumulation assay

The lipofuscin fluorescence in *C. elegans* on the seventh day of L1 stage was measured according to Pincus et al. (Onken and Driscoll, 2010). Worms were anesthetized using 1 M sodium azide and mounted on a 2% agarose pad. Subsequently, the fluorescence intensity of approximately 30 individuals per treatment was determined and imaged using fluorescence microscopy (CX23, Olympus, Tokyo, Japan) and ImageJ software to reflect the accumulation levels of lipofuscin.

### 2.10 Neuroprotective activity

#### 2.10.1 Inhibition of the acetylcholinesterase enzyme

The *in vitro* AChE inhibition activity of the CX extracts was assessed (Wang et al., 2016). Briefly, a reaction mixture containing 20 μl of phosphate buffer (200 mM, pH 7.7), 20 μl extract (1 mg/ml), 80 μl dithiobis nitrobenzoic acid (DTNB) (1 mM), and 30 μl AChE (2 U/ml) was incubated for 20 min at 37°C. After that, 35 μl of 7.5 mM substrate acetylthiocholine iodide (ATCI) solution was added. After incubation for 20 min at 37°C, the absorbance was measured in a 96-well microplate reader at 412 nm. HupA (1 mg/ml) was used as positive control. The AChE inhibitory activity were expressed as percentage inhibition (%), which was calculated using the following formula: [(control absorbance-sample absorbance)/control absorbance] ×100.

The *in vivo* AChE activity of the CX extracts in worms was also determined according to the instructions supplied with the commercial assay kits.

#### 2.10.2 Chemotaxis assay using *C. elegans* CL2355

Chemotaxis assay was performed as previously described (Peixoto et al., 2016) with modifications. L1-stage synchronized CL2122 (no Aβ) or CL2355 (Aβ) worms were incubated with or without CX at 16°C for 36 h and then at 23°C for 36 h. The temperature upshift is required for the neuronal Aβ expression in the mutant strain CL2355. The mutant strain CL2122 was used as a control strain. The collected worms were washed with M9 buffer three times to remove all bacteria. Finally, 30 worms were placed in the center of the 2% agar assay plate (35 mm), which was divided into normal (N) and trap (T) zones (Figure 8A). Before the placement, control odorant (1 μl of 100% ethanol and 1 µl of 1 M sodium azide) and attractant (1 μl of 0.1% benzaldehyde and 1 µl of 1 M sodium azide) were added to a spot about 3 mm away from the plate edge on the N zone and T zone, respectively. The plates were incubated (1 h at 23°C) and the number of worms at the different zones was scored. The chemotaxis index was calculated as (T-N)/(T + N), where N and T represented the number of worms at odorant control and attractant position, respectively.

#### 2.10.3 Paralysis assay

*C. elegans* CL4176 is a temperature-sensitive mutation that expresses human amyloid a*β*_1-42_ in muscle cells when the temperature is increased from 16°C to 25°C, which results in the paralytic phenotype of CL4176s (Wang et al., 2017). L1 stage -synchronized CL4176 worms were cultivated on fresh NGM plates in the presence of CX extract and in the absence of them at 16°C for 36 h. Afterwards, they were incubated at 25°C to induce Aβ transgene expression (Dostal and Link, 2010) and paralysis was scored at every 2 h until all worms were paralyzed (Moliner et al., 2019). For each assay, at least 100 worms were used per condition.

#### 2.10.4 Amyloid-β aggregate staining assay

CL4176 was cultured as described for the paralysis assay and Aβ aggregates were visualized by Thioflavin T staining. First, worms were fixed with fixer solution (4% paraformaldehyde/M9 buffer, pH 7.4) at 4°C. After 24 h, worms were transferred to a solution containing 5% fresh β-mercaptoethanol, 1% Triton X-100 and 125 mM Tris (pH 7.4) for 24 h at 37°C. Next, excess penetrant was removed with M9 and stained with 0.125% Thioflavin T in 50% ethanol for 30 min. Finally, excess Thioflavin T was removed with different concentrations of ethanol washes (50%, 75%, 90%, 75% and 50% v/v). Stained worms were observed using a fluorescence microscope (CX23, Olympus, Tokyo, Japan) and images were acquired at ×40 magnification.

### 2.11 Expression levels of gene (RNA isolation, reverse transcription and qRT-PCR analysis)

Firstly, Total RNA was extracted in N_2_ or CL4176s on the fourth day of egg using the TRIzol Total RNA Extraction Kit (TIANGEN) *via* standard protocol and then reversed transcription into c-DNA by RT reagent kit. The Quantitative Real-Time PCR (qPCR) analysis (CFX96 real-time PCR detection system, Bio-Rad) was conducted with the SYBR green kit. The forward and reverse primer sequences are listed in Supplementary Table S1. The relative mRNA levels with β-actin mRNA as reference genes for normalization, was analyzed using the 2^−ΔΔCt^ method (Peng et al., 2016).

### 2.12 Body length and brood size assay

The young adult worms were transferred to 2% agarose pads on slides and anesthetized with 1 M sodium azide. The bright field image of at least 30 worms per group was captured using a confocal fluorescence microscopy (Carl Zeiss, Jena, Germany). The body size was analyzed using ImageJ software.

Determination of the effects of CXL and CXR on fecundity according to the method of Dulovic et al. (2016). Every synchronized worm (pre-treated with CXL and CXR) at L4 began to transferred to a fresh NGM plates every 24 h and allowed to lay eggs during the breeding period. The Eggs were allowed to hatch into the L1-4 larval stage for easy observation and the total offspring produced by each individual was counted per day and finally summed up. The test was performed three times and about 10 worms in each group.

### 2.13 Statistical analysis

Data are displayed as means ± SD and analyzed by SPSS 24.0 of one-way ANOVA test followed by Duncan’s multiple comparison test. The survival curve analyzed by GraphPad Prism six software and assessed using the Kaplan–Meier log-rank test. Other figures were plotted by origin.20. Any two groups marked with different letters in the histogram represent statistically significant differences, and *p*-value was *(*p* < 0.05), **(*p* < 0.01), and ***(*p* < 0.001) was considered statistically significant.

## 3 Results

### 3.1 Identification of compounds in CXL and CXR

**FIGURE 1:**
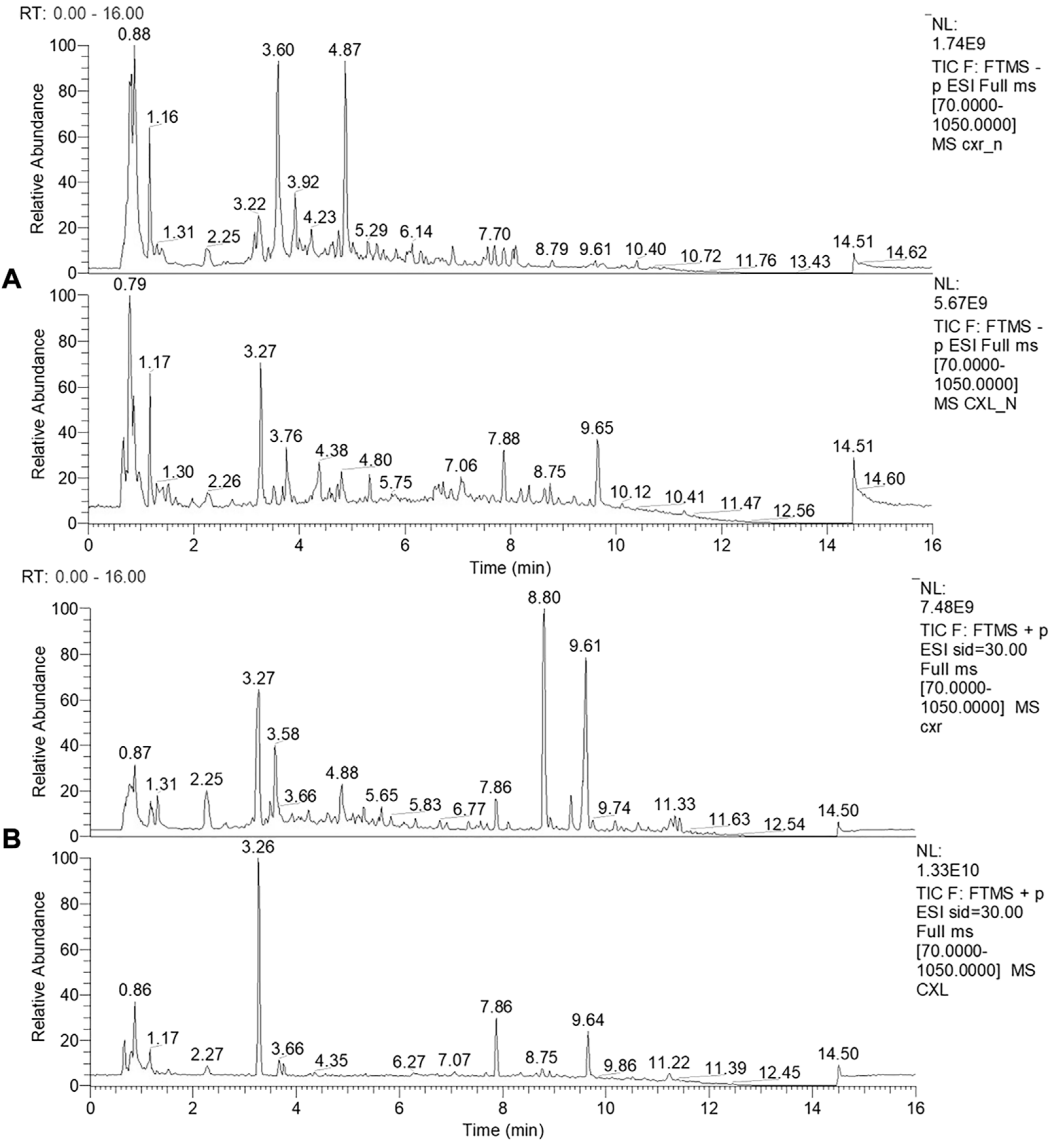
LC-MS profile of CXR and CXL. **(A)** LC-MS profile of CXR and CXL in negative ionization modes. **(B)** LC-MS profile of CXR and CXL in positive ionization modes.

In this work, the CXL and CXR composition was identified by LC-MS. Representative chromatograms of the CXL and CXR in positive and negative ionization modes are shown in Figure 1. In each analytical replicate, approximately 500 mass spectrum outputs were examined, resulting in the tentative identification of 98 compounds, including phthalides (1–4), alkaloids (5–17), organic acids (18–39), terpenes (40–43), polyphenols (44–78) and others (79–98). In Supplementary Table S2, the identified compounds are listed with their corresponding Rt, observed (m/z), [M-H] ^−/+^, molecular formula, and MS/MS fragments. Among 98 identified compounds, 74 compounds were identified in CXR, and polyphenols were the main compounds, including phenolic acids, flavonoids, anthocyanins and simple phenols. In addition, 4 phthalides, 11 alkaloids, 15 organic acids and 3 terpenes were also detected, most of which have been found in *Chuanxiong* (Chen et al., 2018). Compared with CXR, 78 compounds were identified in CXL, which contains more polyphenolic compounds and organic acids and less alkaloids and phthalides. Overall, these results provided a database for structural analysis of active compounds involved in health benefits.

### 3.2 *In vitro* antioxidant activity

**FIGURE 2:**
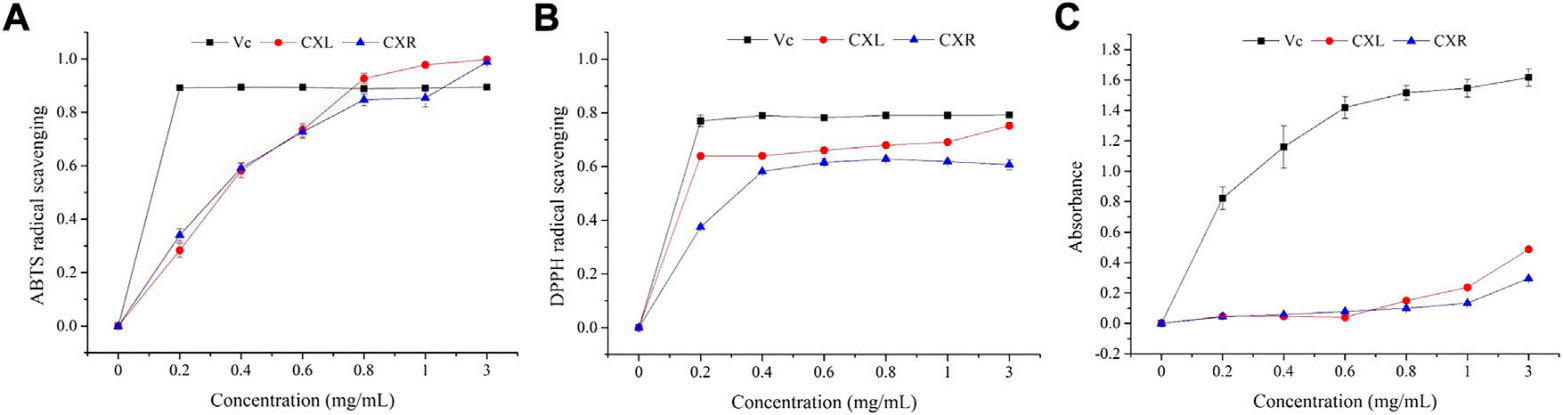
Antioxidant activities of CXL and CXR with six gradient concentrations (0.0, 0.2, 0.4, 0.6, 0.8, 1.0, and 3.0 mg/ml). **(A)** ABTS radical scavenging activity. **(B)** DPPH radical scavenging activity. **(C)**Reducing power. Vc was used as the positive control.

The CXL and CXR *in vitro* antioxidant potential was analyzed on the basis of radical scavenging and reducing power assay. Figure 2 shows that two sample concentrations ranged from 0.2 to 3 mg/ml, and that antioxidant activity varied dose-dependently. Both extracts showed a similar IC_50_ values of ABTS scavenging activity is 0.325 ± 0.013 for CXL and 0.310 ± 0.011 mg/ml for CXR (Table 1). In the DPPH radicals scavenging activity assay, the IC_50_ values of CXL and CXR extracts are 0.018 ± 0.011 and 0.269 ± 0.009 mg/ml, respectively, indicating that CXL extracts is with higher *in vitro* antioxidant activity when compared to CXR extracts (*p* < 0.05). Additionally, CXL extracts showed similar DPPH radical scavenging activity to VC standard (*p* > 0.05; Table 1). However, both extracts at all concentrations had the lower FRAP values as compared to that of the reference (VC) (Figure 1). In accordance with the antioxidant activities, higher phenolic and flavonoid contents of 33.22 ± 3.65 mg GAE/g dry weight sample and 20.23 ± 2.37 mg RE/g dry weight sample were determined from the CXL extract, respectively (Table 1).

**TABLE 1:** Total phenolic content, total flavonoid content and free radical scavenging capacity of CXL and CXR.

Extract	Total phenolics mg GAE/g	Total flavonoids mg RE/g	DPPH scavenging assay	ABTS scavenging assay
			IC_50_ value (mg/ml)	
CXL	33.22 ± 3.65_a_	20.23 ± 2.37_a_	0.018 ± 0.011_b_	0.325 ± 0.013_a_
CXR	26.02 ± 3.41_a_	13.31 ± 0.98_b_	0.269 ± 0.009_a_	0.310 ± 0.011_a_
Vc			0.017 ± 0.008_b_	0.03 ± 0.007_b_

Different letters indicated statistical significance (*p* < 0.05).

### 3.3 Effect of CXL and CXR on acute toxicity in N_2_ *C. elegans*

The acute toxicity of CXL and CXR on *C. elegans* could be reflected by viability. According to the result in Supplementary Table S3, a range of concentrations between 250 and 1,000 g/ml of CXL and CXR were found to be safe for *C. elegans*, which did not affect worm viability after 24 h when compared to the control group.

### 3.4 Effect of CXL and CXR on oxidative stress in N2 *C. elegans*

**FIGURE 3:**
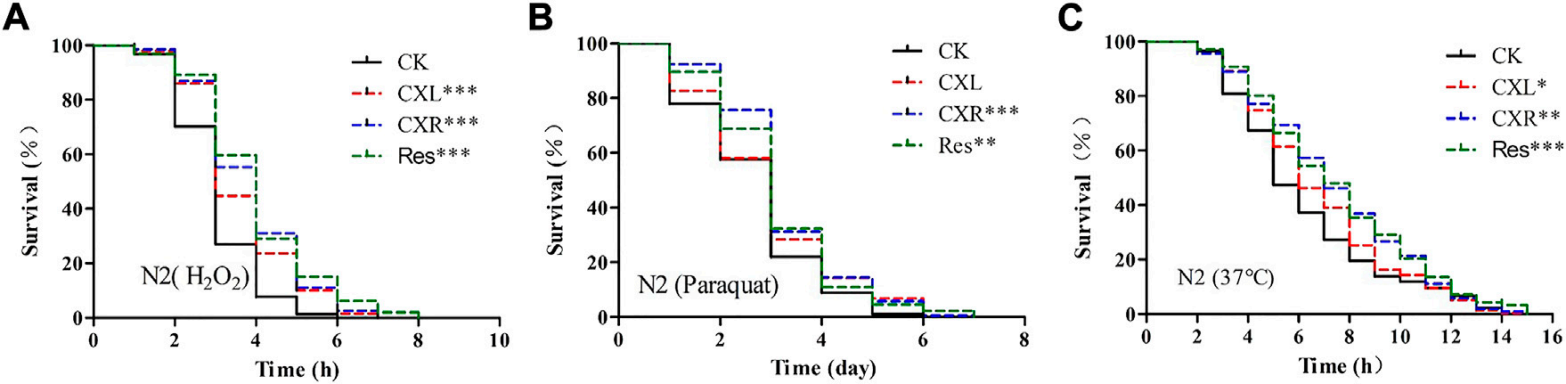
Effect of CXL and CXR on stress resistance in *C. elegans*. **(A)** Survival curve of the N_2_ strain under H_2_O_2_-induced oxidative stress. **(B)** Survival curve of the N_2_ strain under paraquat-induced oxidative stress. **(C)** Survival curve of the N_2_ strain under heat shock stress. Res was used as the positive control. Three independent biological replicates were performed. Significant differences were determined by a log-rank test, with *p < 0.05; **p < 0.01 and ***p < 0.001.

Oxidative stress was induced in *C. elegans* by exposure to H_2_O_2_ or paraquat, which are strong natural pro-oxidant. Firstly, we found that CXL and CXR treatment promoted a significant right shift in the survival curve under H_2_O_2_- induced oxidative stress when compared with the control (Figure 3A; *p* < 0.001), and increased the mean survival time by 23.32% and 31.62%, respectively (Supplementary Table S4). Moreover, only the survival rate of CXR treatment was consistently higher than that of the control group (*p* < 0.001) while CXL treatment had no significant effect on the survival rate under the paraquat-induced oxidative stress (Figure 3B; Supplementary Table S4; *p* > 0.05). Therefore, the stress tolerance of CXL ability under H_2_O_2_-induced oxidative stress was more stable than that under paraquat-induced oxidative stress. Secondly, thermotolerance was further evaluated, pretreatments with the extracts had a positive impact on the response of worms under heat stress condition by increasing the survival rate in relation with a control group (Figure 3C). CXL and CXR-treated worms showed a 13.04% and 19.38% significant increase in mean lifespan compared to untreated control worms, respectively (Supplementary Table S4; *p* < 0.05), thereby indicating that CXL and CXR conferred heat and oxidation stress resistance in *C. elegans*.

### 3.5 Effect of CXs on the accumulation of ROS and MDA and the activities of SOD and GSH-PX in N2 *C. elegans*

**FIGURE 4:**
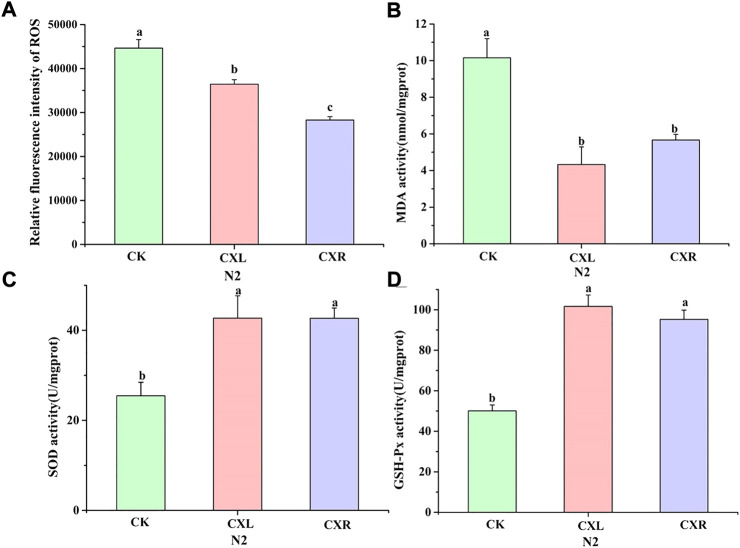
Effect of CXL and CXR on the antioxidant defense system in *C. elegans.* **(A)** The accumulation of ROS. **(B)** The content of MDA. **(C,D)** The activity of GSH-PX and SOD. Bars with different letters indicated statistical significance (*p* < 0.05).

We could intuitively see that ROS production levels were visibly reduced after CXL and CXR intervention (Figure 4A; *p* < 0.05), suggested it could scavenge the overproduction of ROS. Furthermore, MDA is one of the most important products of membrane lipid peroxidation, which can cause damage to membrane system. As shown in Figure 4B, a marked reduction of MDA was observed in CXL and CXR treated group (*p* < 0.05). What’s more, SOD is a natural superoxide radical scavenging enzyme in animals and plants. Our results showed that the activity of SOD treated with CXL and CXR was 42.49 ± 4.98 U/ml and 42.68 ± 2.28 U/mL, and increased by 67.67% and 67.64%, respectively, when compared with the control group, (Figure 4C). In addition, GSH-PX activity was obviously increased after CXL and CXR exposure (Figure 4D). Besides, we noticed that the activity of GSH-PX and SOD and the content of MDA and ROS were not significantly changed between CXL and CXR treatment group except ROS content (*p* > 0.05). In brief, CXL and CXR intervention had effectively increased antioxidant enzyme activity to alleviated MDA and ROS accumulation, which acts as an antioxidant.

### 3.6 Effect of CXL and CXR on the lifespan of N2 *C. elegans*

**FIGURE 5:**
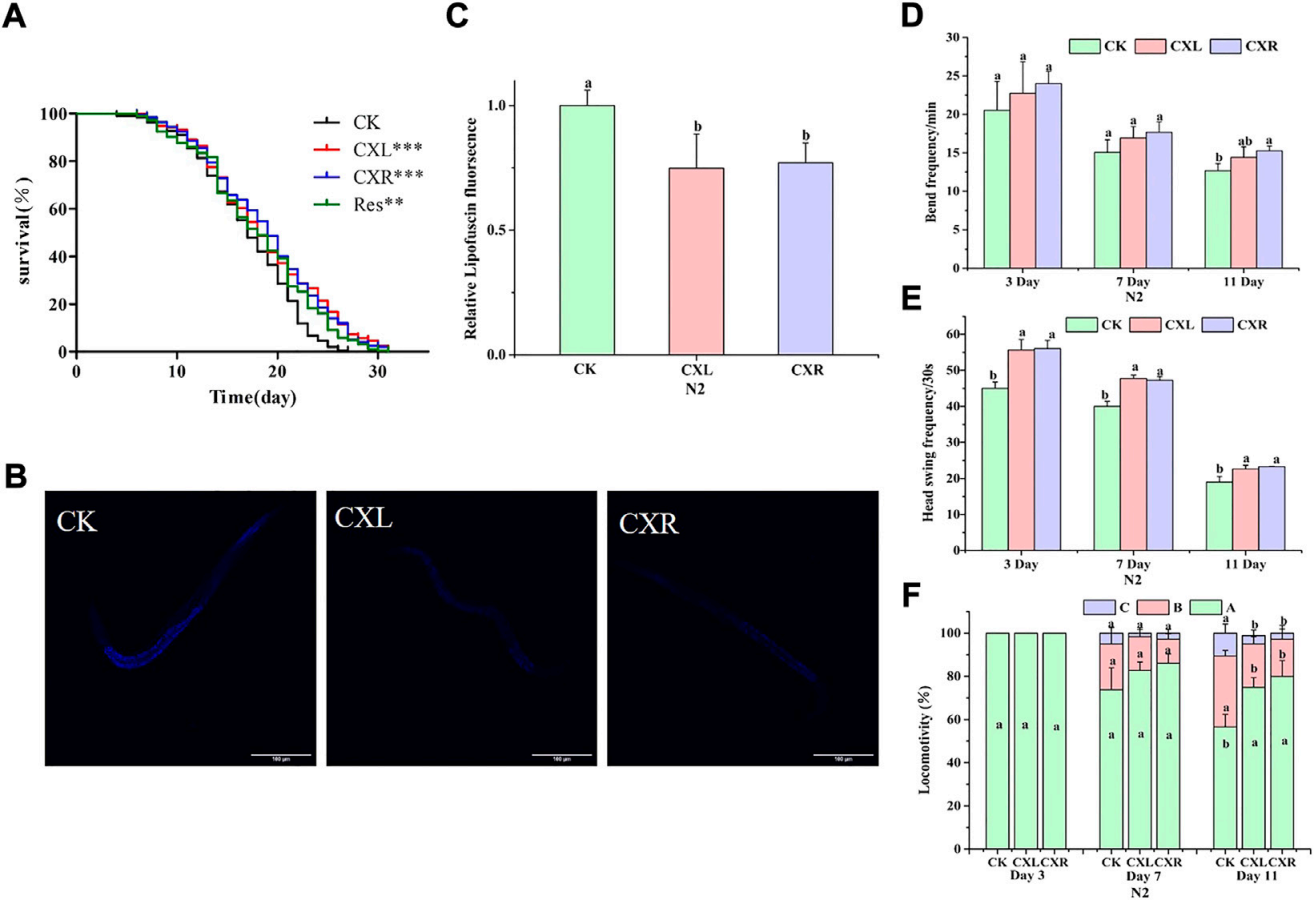
Effect of CXL and CXR on lifespan and healthspan of *C. elegans*. **(A)** Lifespan. **(B)** Representative pictures of lipofuscin in worms treated with CXL and CXR. **(C)** The content of lipofuscin was quantified according to ImageJ software. **(D)** The frequency of the body bends. **(E)** The frequency of the head swing. **(F)** The three levels of locomotivity were measured, A-free movement, B-movement after prodding, C-weak movement after prodding. Bars with different letters indicated statistical significance (*p* < 0.05). Res was used as the positive control.

The lifespan properties of CXL and CXR were determined using wild-type worms exposed to CXL and CXR extracts at 20°C. Figure 5 shows a significant right shift in the survival curve in CXL and CXR treatment group compared to the control group and the protective effect of CXL and CXR on delaying aging becomes evident in this assay (Figure 5A; *p* < 0.0001). However, the two extracts did not significantly affect the median time and maximum time (*p* > 0.05), while the mean time has increased significantly (Supplementary Table S4; *p* < 0.05). Our results clearly revealed that CXL and CXR extracts significantly prolonged the lifetime of the worms.

### 3.7 Effect of CXL and CXR on the healthspan of N2 *C. elegans*

#### 3.7.1 Movement assay

The viability of *C. elegans* is indirectly reflected by the move ability during a certain period of time and responds to external mechanical stimuli. We assessed motor ability at the early, middle, and late stages of life (on day 3, 7, and 11), using three indicators: body bend, head swing, and locomotion. The worms treated with CXL and CXR exhibited the greatest improvement in the head swing frequency and movement ability, especially in the mid-late stages (Figures 5E,F). Moreover, a significant increase in body bend frequency was also observed on day 11 for worms treated with CXR (Figure 5D). These results suggest that CXL and CXR treatment resulted in a positive impact on movement at different ages of adulthood.

#### 3.7.2 Lipofuscin accumulation assay

The accumulation of lipofuscin is another important feature of health or aging. Therefore, we also evaluated lipofuscin accumulation in *C. elegans*. The results showed that the accumulation of lipofuscin in CXL and CXR treatment groups decreased by 0.26% and 0.23%, respectively, compared with the control group (*p* < 0.05; Figures 5B,C). This phenomenon indicated that CXL and CXR could decrease the accumulation of lipofuscin.

### 3.8 Effect of CXL and CXR promotes longevity and stress resistance of *C. elegans via* daf-16, daf-16 and hsf-1 to activate related genes, respectively

**FIGURE 6:**
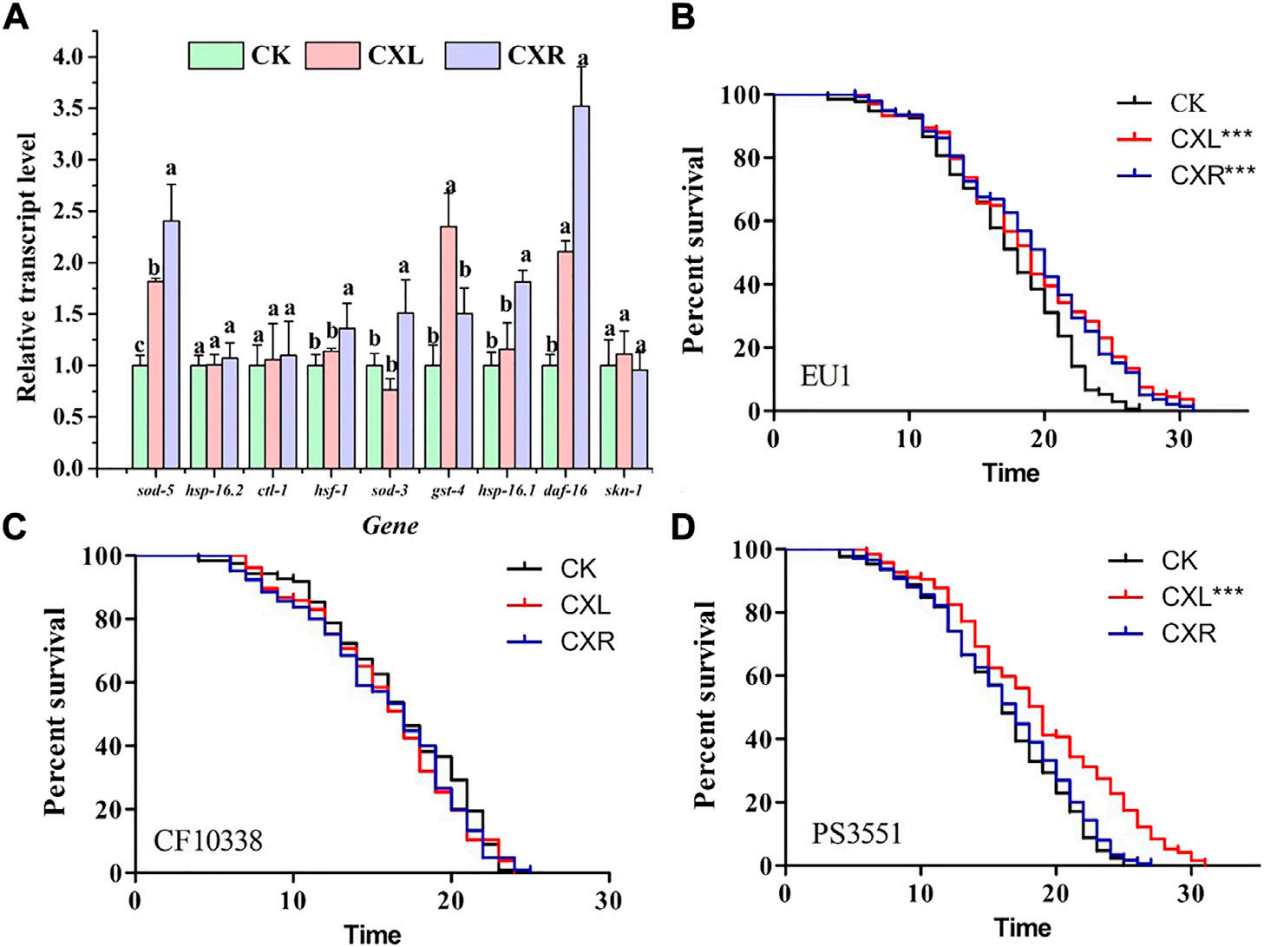
The molecular mechanism of CXL and CXR in the anti-aging. **(A)** The expression level of age-related genes in *C. elegans*. **(B)** The survival curve of *skn-1* mutant worms. **(C)** The survival curve of *daf-16* mutant worms. **(D)** The survival curve of *hsf-1* mutant worms. Bars with different letters indicated statistical significance (*p* < 0.05). Significant differences were determined by a log-rank test, with *p < 0.05; **p < 0.01 and ***p < 0.001.

To investigate the molecular mechanisms of CXL and CXR biological effectors, RT-qPCR method was used to evaluate their effects on the expression of genes related to aging or stress response, such as *daf-16*, *gst-4*, *ctl-1*, *skn-1*, *hsf-1*, *hsp-16.2*, *hsp-16.1, sod-3*, and *sod-5*. As shown in Figure 6A, neither CXL nor CXR treatments significantly changed the expression levels of *skn-1*, *hsp-16.2* and *ctl-1*, while both extracts up-regulated the expression levels of *daf-16* and *sod-5*. Moreover, we found that CXR and CXL elevated the expression of the *hsf-1*, *hsp-16.1*, *sod-3*, and *gst-4* genes, respectively. Taken these data together, the overexpression of those genes suggests that they participated in CX’ antioxidant and anti-aging effects in N_2_ *C. elegans*. Neither CXL nor CXR treatment affected the lifespan of mutants compared to the control group (Figure 6B).

Furthermore, we found no significant increase in survival of CXR-treated daf-16 and hsf-1 mutants compared to controls (Figures 6C,D, *p* > 0.05), proving that CXR may be dependent on *daf-16* and *hsf -1* instead of skn-1. Only daf-16 mutants did not show the protective effect of CXL on longevity in worms (Figure 6C; *p* > 0.05), suggesting that daf-16 may be required for CXL to promote longevity.

### 3.9 Effect of CXL and CXR on the inhibition of the AChE

**FIGURE 7:**
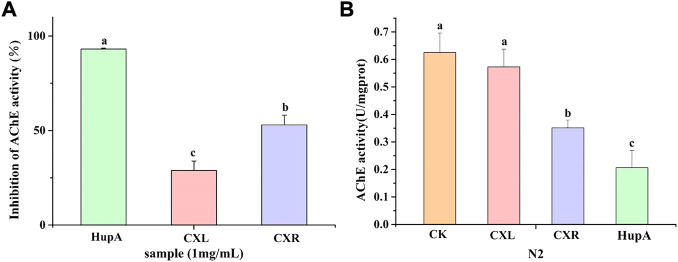
Effect of CXL and CXR on the AChE inhibition activity. **(A)** AChE inhibition activity of the CXL and CXR *in vitro*. **(B)** AChE activity of the CXL and CXR in worms. HupA was used as positive control. Bars with different letters indicated statistical significance (*p* < 0.05).

The ability of the CXL and CXR to inhibit acetylcholinesterase (AChE) activity *in vitro* was evaluated and the result is shown in Figure 7A. The result revealed that both extracts show inhibition against AChE, but significantly lower than HupA at the same concentration. Also, a remarkable variation was observed among the extracts and the prominent inhibitory effects against AChE (52.99 ± 5.16%) was caused by the CXR extract (Figure 7A).

The CXL and CXR extracts were tested for their capacity to inhibit AChE *in vivo* using *C. elegans*. Figure 7B shows the AChE activity of only the CXR treatment group was significantly lower than the control group. Although not as effective as positive controls, CXR might effectively inhibit this enzyme compared with the control group.

### 3.10 Effect of CXL and CXR on aβ-induced chemotactic dysfunction in *C. elegans* CL2355

**FIGURE 8:**
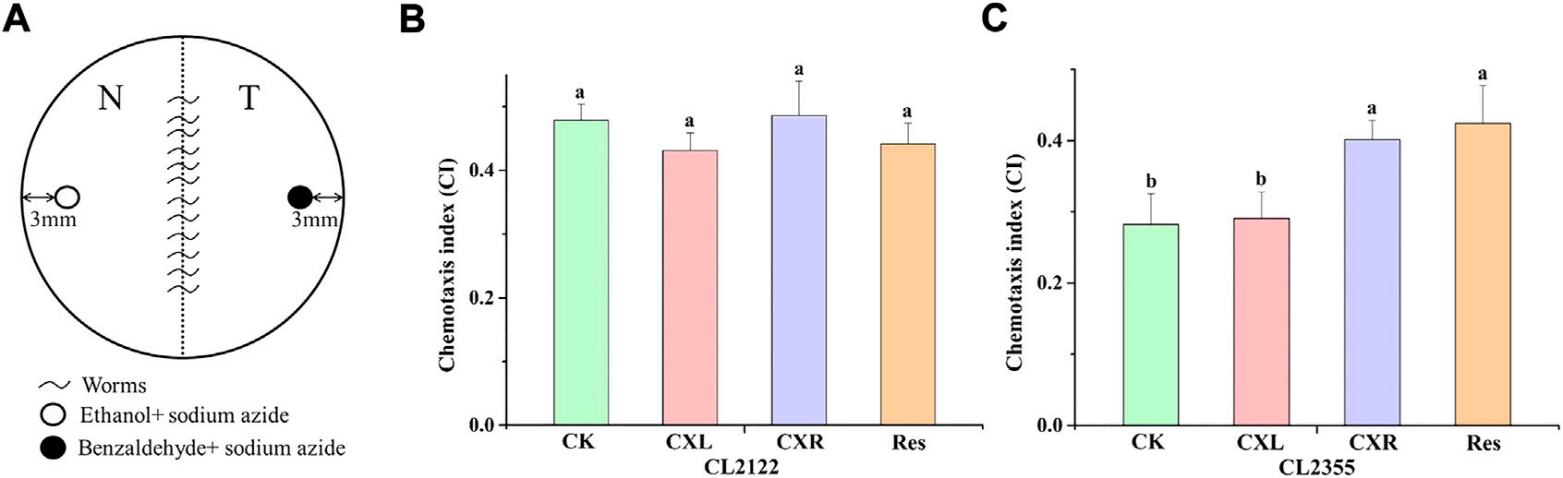
Effect of CXL and CXR on Aβ-induced chemotactic dysfunction in *C. elegans*. **(A)** The schematic of chemotaxis assay. **(B)** The chemotaxis index after treatment of CL2122 worms with CXL or CXR. **(C)** The chemotaxis index after treatment of CL2355 worms with CXL or CXR. Bars with different letters indicated statistical significance (*p* < 0.05).

Benzaldehyde is an attractants of worms, attracting healthy worms and crawling towards benzaldehyde, while chemotactic dysfunction worms are not attracted. Therefore, we determined the alleviating effects of CXL and CXR on Aβ-mediated neurotoxicity using the *C. elegans* CL2355, which expresses Aβ and causes chemotactic dysfunction (Kampunzu et al., 2009). As shown in Figure 8B, the chemotaxis indexes of the CL2122 (no Aβ expression) and the untreated Aβ strain CL2355 were 0.48 and 0.28, respectively, demonstrating more behavioral disorders in CL2355 worms. Meanwhile, CXL and CXR did not affect the chemotactic behavior of worms CL2122 (Figure 8B). When compared to the untreated CL2355, the chemotaxis index of CXR-treated CL2355 worms was significantly increased by 42.96% (Figure 8C). It is shown that CXR has a protective effect against Aβ-mediated neurotoxicity by observing the chemotactic behavior of worms.

### 3.11 Effect of CXL and CXR on aβ-induced paralysis in *C. elegans* CL4176

**FIGURE 9:**
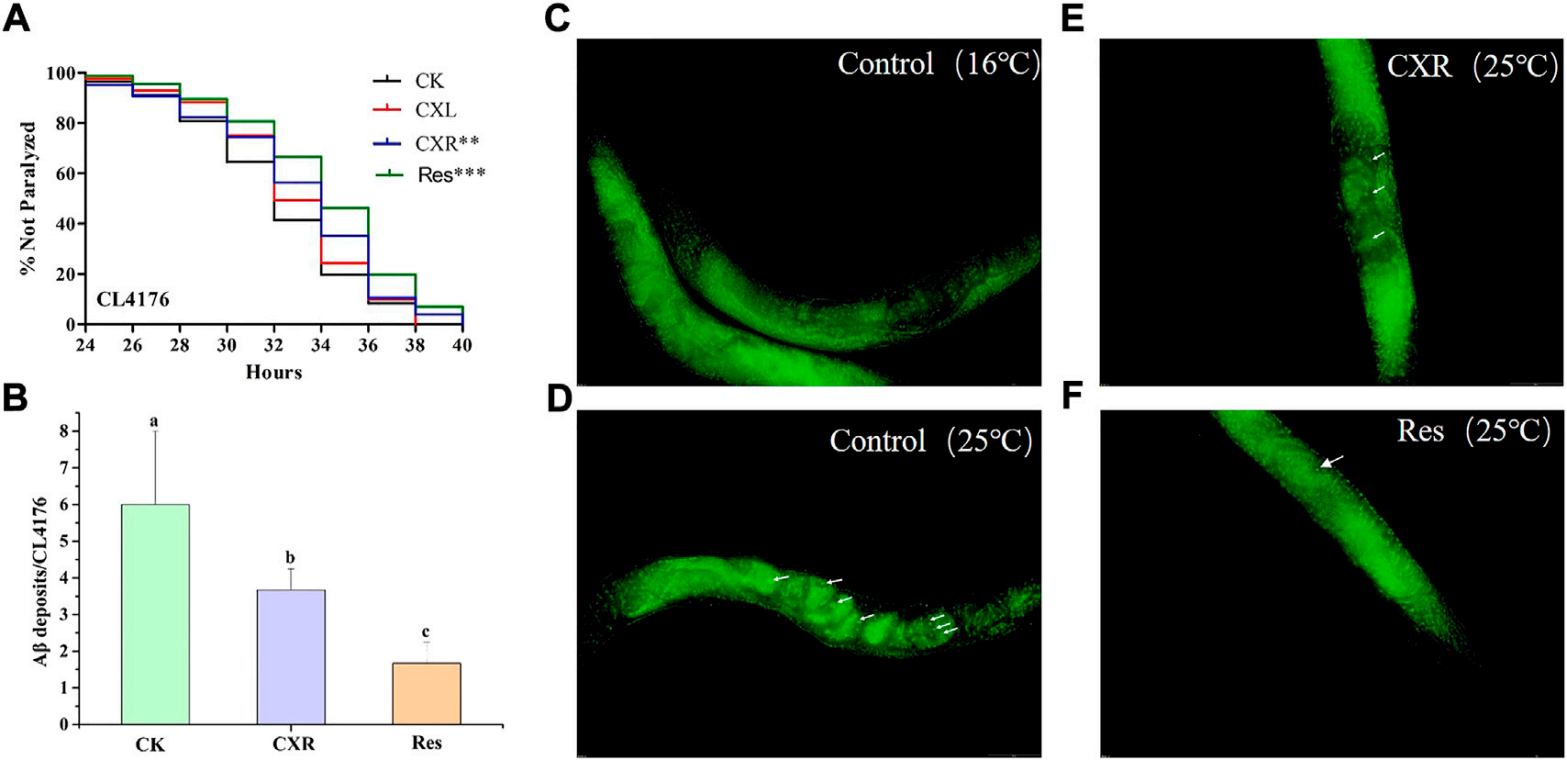
Effect of CXL and CXR on Aβ-induced paralysis phenotype in CL4176 *C. elegans*. **(A)** Paralysis curve shown non-paralyzed CL4176 worms after the CXL and CXR treatment. **(B)** Statistics of the number of Aβ deposits in nematodes from the different groups. **(C)** Thioflavin T staining images of Aβ deposition in CL4176 *C. elegans* that were maintained at a permissive temperature (16°C) were used as negative controls. **(D)** Thioflavin T staining images of Aβ deposition in CL4176 *C. elegans* after the increase of temperature. **(E)** Thioflavin T staining images of Aβ deposition in CL4176 *C. elegans* after treatment with CXR. **(F)** Res was used as a positive control. The Fluorescence images were observed by a fluorescent microscope at ×40 magnification. Aβ aggregates are remarked with white arrow. Bars with different letters indicated statistical significance (*p* < 0.05). Significant differences were determined by a log-rank test, with *p < 0.05; **p < 0.01, and ***p < 0.001.

Aβ is well-known as a neurotoxic agent which can cause neurological diseases (Chen et al., 2015). CL4176s is a model for screening neuroprotective substances due to its ability to promote the expression and accumulation of A*β*_1-42_ in the muscle tissue by raising the ambient temperature and eventually paralyzes (Link et al., 2003). As shown in Figure 9A, the paralysis phenotype was reduced with CXR treatment, which exhibited significantly different right-shifted paralysis curves compared with the control group (*p* < 0.01). But CXL did not increase the mean time of paralysis of CL4176 worms (*p* > 0.05). Furthermore, the paralysis time for 50% of worms (PT_50_) was calculated in Supplementary Table S5, the CXR delayed significantly the PT_50_ by around 7.47%. However, the PT_50_ was not different between treatment groups of CXL and control. These findings demonstrated that CXR possesses neuroprotective potential by alleviating β-amyloid toxicity in the *C. elegans* AD model.

### 3.12 Effect of CXR treatment on aβ accumulation in *C. elegans* CL4176

Since CXR can prolong the paralysis time of CL4176s, it was selected to further study the possible mechanism of alleviating paralysis symptoms. Thioflavin T staining showed that the amount of Aβ deposited in CL4176s increased significantly when the temperature increased from 16°C (Figure 9C) to 25°C (Figure 9D). In contrast, the CXR treatment group was significantly lower than that in the control group at 25°C (Figures 9E, F), which demonstrated that CXR treatment decreased Aβ production and/or accumulation to relieve paralysis in CL4176s (Figure 9C).

### 3.13 Effect of CXR treatment on oxidative stress and expression of related genes in *C. elegans* CL4176

**FIGURE 10:**
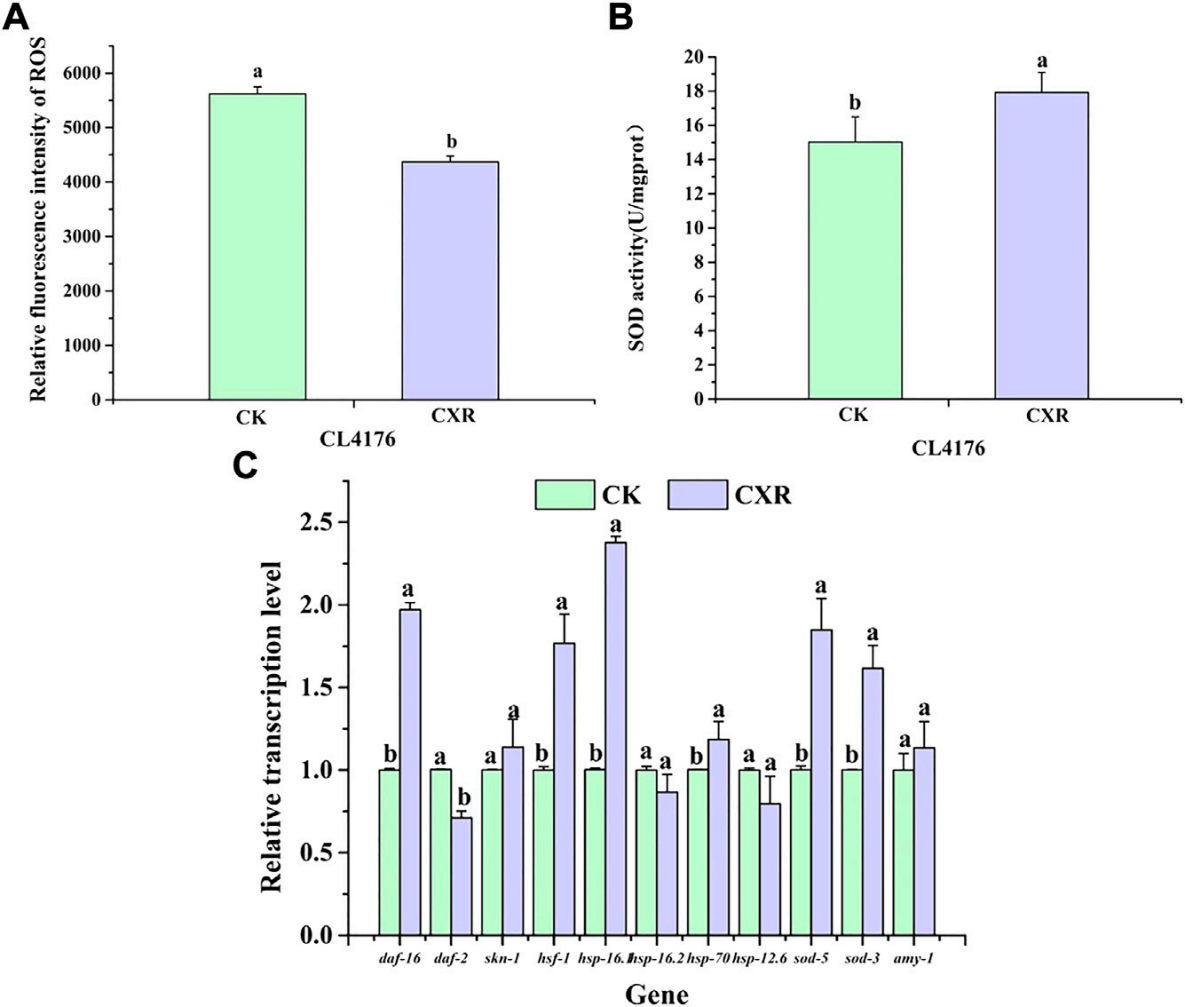
Effect of CXR on oxidative stress and expression of related genes in CL4176 *C. elegans*. **(A)** The ROS was measured in worms from the different groups at 36 h after temperature uplift to 25°C using 2′, 7′-dichlorofluorescein diacetate. **(B)** The SOD activity in worms from the different groups. **(C)** The relative expression of related genes was calculated using the method of 2^−ΔΔCt^ and the gene *act-1* was used as the internal reference. Bars with different letters indicated statistical significance (*p* < 0.05).

Next, we investigated the effect of CXR on ROS accumulation using fluorescence methods. As shown in Figure 10A, ROS was reduced by approximately 22% in the CXR-treated group when compared to the control group at 36 h after the temperature increase. In addition, considering that SOD-3 is one of the important ROS scavenging enzymes in the antioxidant enzyme system, it can catalyze the conversion of active superoxide anion to molecular oxygen. We found that CXR treatment significantly increased SOD activity in CL4176s (Figure 10B; *p* < 0.05)).

To further explore the possible molecular mechanism of CXR in alleviating paralysis symptoms, we selected genes of interest for qPCR. First of all, previous research has found that Skn-1, HSF-1, and Daf-16 play crucial roles in regulating Aβ aggregation (Guo et al., 2016). As shown in Figure 10C, in the CXR treated CL4176s, the expression levels of *daf-16* and *hsf-1* were increased by 97%, and 77% respectively, compared to the control (*p* < 0.05), while the expression level of *daf-2* was significantly down-regulated and the expression levels of *skn-1* was not significant between the treatment group and control group. Next, since expression levels of these transcriptional factors (*daf-16* and *hsf-1*) were significantly increased, we further tested the expression of their downstream genes. The results showed that compared to the control, CXR-treated CL4176s significantly upregulated the expression of *hsp-16.1*, *hsp-70*, *sod-5*, and *sod-3*. Finally, there is no significant difference in the expression levels of *amy-1* between the CXR treatment group and the control group.

### 3.14 Effect of CXL and CXR on the growth and fertility of N_2_ *C. elegans*

**FIGURE 11:**
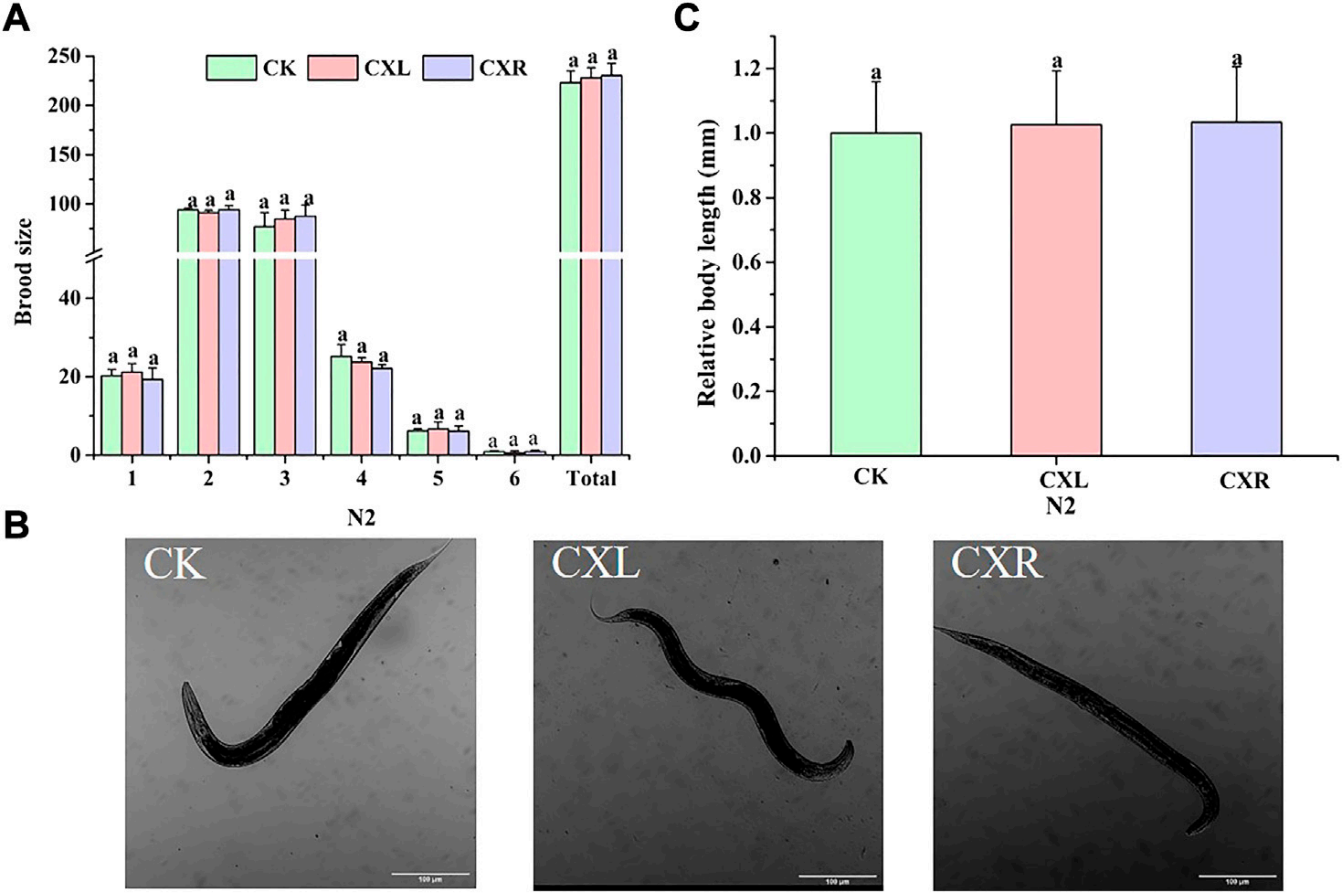
Effect of CXL and CXR on the body size and reproduction of **(C)** *elegans*. **(A)** Brood size. **(B)** Representative picture of body length of **(C)** *elegans*. **(C)** Body length was quantitated by image J. Bars with different letters indicated statistical significance (*p* < 0.05).

To determine whether the extract at this dose has adverse effects on *C. elegans* physiological functions, we evaluated the effects of CXL and CXR on worm reproduction and body size. We monitored the size of the brooding per day and found that CXL and CXR were not able to provoke any changes compared to the control group, neither in daily brood size nor total brood size (Figure 11A). Additionally, there were no significant differences in body length between CX-treated groups and control groups (Figures 11B,C). Taken together, CXL and CXR might effectively increase lifespan and decrease proteotoxic without causing obvious side effects in the reproduction and growth of *C. elegans*.

## 4 Discussion

*Ligusticum chuanxiong* Hort. (CX) widely used as a health food product or drug for the health benefits including protection against headache, cerebrovascular, cognitive and motor deficits diseases (Chen et al., 2018; Liu et al., 2018). Those effects could be attributed to their active ingredient, such as ferulic acid (Zhang et al., 2018), ligustrazine (Jiang et al., 2022) and ligustilide (Huang et al., 2013) et al. At present, a large number of studies focus on the tuber of *Ligusticum chuanxiong*, but ignore the study of *Ligusticum chuanxiong* leaf. In this study, LC-MS analysis confirmed that both CXL and CXR extracts contain quite a few phytochemicals, including phthalides, alkaloids, organic acids, terpenes, and polyphenols. CXR contains relatively rich alkaloids, phthalides and terpenes, while the CXL contains rich polyphenols and organic acid compounds, which suggests that in addition to *chuanxiong* rhizome, *Chuanxiong* leaves also is a potentially abundant source of phytochemicals. Based on these data, we can correlate the results observed in present study with the chemical constituents identified in the extract. However, compared with previous studies (Yan et al., 2022), CXL and CXR showed higher radical scavenging capacity. These differences can be attributed to differences in geography, harvest time, storage methods, and different extraction procedures and solvents. Notably, CXL has stronger free radical scavenging ability than CXR, which may be related to the difference of metabolites in different anatomical parts of plants. In general, the aerial parts of plants are susceptible to biotic or abiotic stress, such as pests, herbivores, *etc.* Therefore, it is necessary to produce a higher level antioxidant metabolite for prevention, such as such as polyphenols and flavonoids (Yan et al., 2022).

Several studies have shown that compounds and extracts derived from edible medicinal plants containing polyphenols can enhance longevity and health in a wide range of organisms (Jiang et al., 2021). In the present study, CXL and CXR increased the mean lifespan of *C. elegans* by 5.8% and 8.4%, respectively, compared with the positive control (Figure 5A), and reached levels of activity comparable to those observed in crude extracts of some medicinal and edible plants such as such as a *Tagetes erecta* L extract and *V. cornuta* extract (Moliner et al., 2018, Moliner et al., 2019). In addition, previous studies have reported anti-aging active ingredients from traditional chinese medicine plant can be generally divided into flavonoids, saponins, polysaccharides, alkaloids, and others, and the anti-aging potential of extracts may be attributed to the synergic and additive action among multiple secondary metabolites (Shen et al., 2016; Wang et al., 2018). In addition, The antioxidant and anti-aging effects of some phenolic acid (ferulic acid, caffeic acid, and chlorogenic acid) or flavonoids (epicatechin, genistein) have been reported (Shen et al., 2016; Pang et al., 2017; Si et al., 2019). Therefore, we propose that the anti-aging activity of CXL or CXR may be related to interactions among various compounds. However, characterization of main functional chemical constituents and these interactions and requires further study.

The free radical theory of aging proposes that ROS, as a by-product of aerobic metabolism, can lead to the accumulation of oxidative stress and accelerate the development of aging (Sohal et al., 2002). Numerous studies have shown that improvements in lifespan and healthspan in *Caenorhabditis elegans* are closely related to enhanced stress resistance (Xiong et al., 2021). In the present study, *C. elegans* tolerance to abiotic stress (35°C-induced heat stress and paraquat-induced oxidative stress) was enhanced in CXL and CXR treated groups (Figure 3). In addition, CXL or CXR significantly reduced ROS and MDA levels and promoted antioxidant enzyme activities such as SOD and GSH-PX (Figure 4), suggesting that the antioxidant activities of CXL and CXR may play an important role in their anti-aging potential.

**FIGURE 12:**
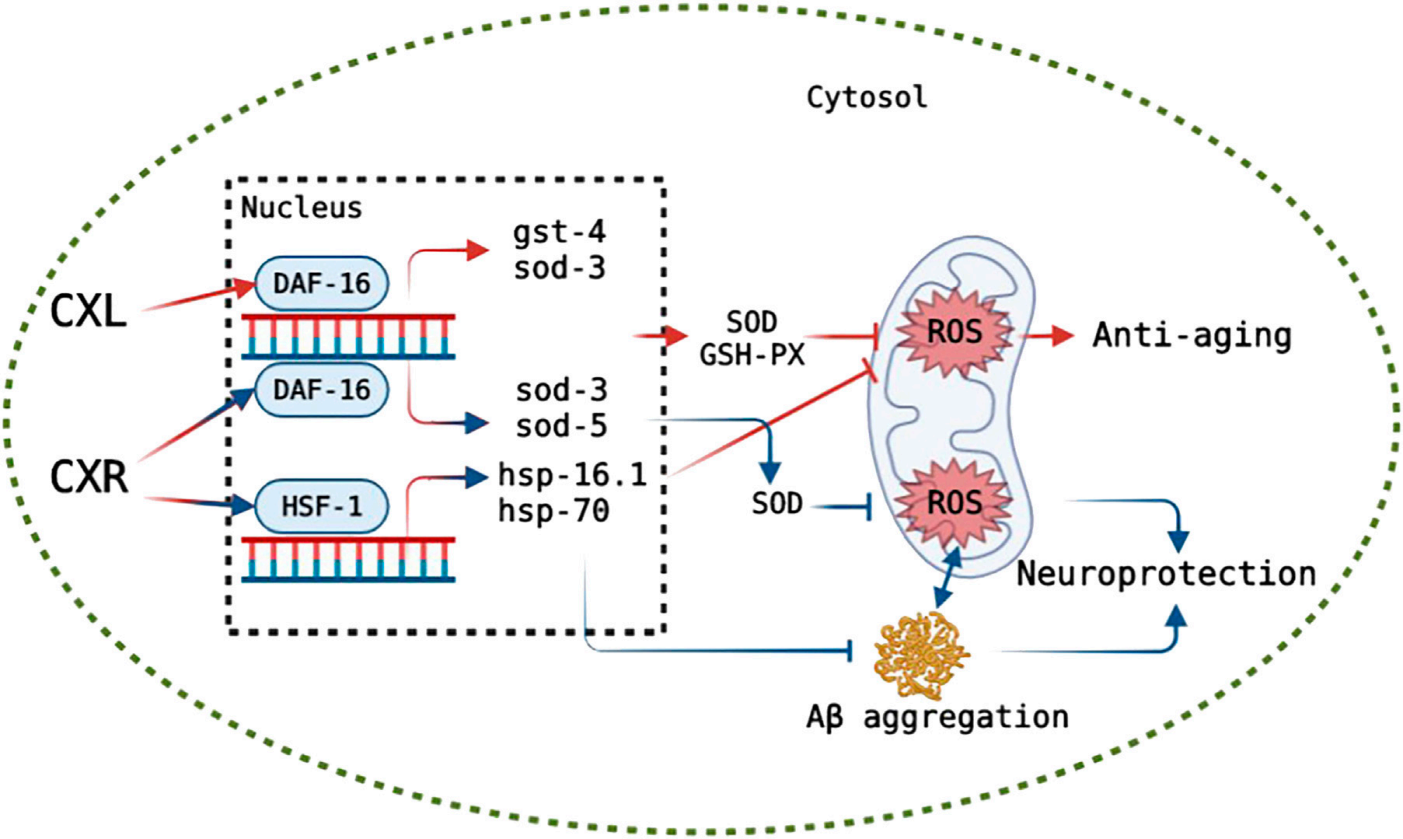
Hypothetical model of the mode of action of CXR and CXL on anti-aging and CXR on neuroprotection. The green circle represents cytomembrane. After entering the cytomembrane, CXL and CXR likely down-regulates ROS level *via* up-regulating DAF-16 or HSF-1 (only in CXR), which in turn likely activates antioxidant oxidase-related and heat shock protein genes expression, respectively, consequently improving the lifespan. CXR alleviates ROS and Aβ accumulation likely *via* activating DAF-16 or HSF-1 that promote downstream expression of related genes, and reduction in ROS level inhibits aggregation of Aβ, consequently displaying neuroprotective potential. Truncated lines indicate a decrease in the process. Lines with arrow indicate a increase in the process. Red: a possible mechanism of CXs on anti-aging. Blue: a possible mechanism of CXR on neuroprotection.

Although our results show that both CXL and CXR improve healthspan and lifespan, the underlying molecular mechanisms warrant further investigation. Thus, the effect of CXR and CXL on the transcriptional expression of some key genes and the longevity of single gene mutants were examined. In *C. elegans*, the transcription factor DAF-16, encoded by daf-16 gene, is a vital regulator in the insulin/IGF-1 signalling pathway and regulates the expression lever of various genes such as *sod-3*, *sod-5*, and *ctl-1*, and it is essential for stress resistance and longevity regulation in (Zei and Braeckman, 2020). In addition to DAF-16 signaling, SKN-1 also contributes to longevity and antioxidant activity by mobilizing a conservative phase 2 detoxification response and activating the expression of several genes, including *gst-4* (Tullet et al., 2008). As another important gene, *hsf-1* encodes the thermal shock transcription factor HSF-1 that regulates the expression of several molecular chaperones (HSP-16.1 and HSP-16.2) in response to thermal and oxidative stress (Kumsta et al., 2017). In the current study, CXR increased the expression of various aging and antioxidant-related genes (*daf-16*, *sod-3*, *sod-5, hsf-1*, and *hsp-16.1*) in *C. elegans* and only skn-1 mutant manifested a significant survival curve after CXR treatment, indicating that CXR prolonged the lifespan of *C. elegans* by activating expression of antioxidant defense system enzyme gene *via* daf-16 and hsf-1 but not skn-1. However, the expression levels of *daf-16*, *sod-3*, and *gst-4* were also up-regulated after CXL treatment and only *daf-16* mutant did not display a significantly different survival curve compared with control worms indicating that CXL-mediated longevity was dependent on daf-16. The findings provided evidence that CXL or CXR improve lifespan in part by activating some important genes in antioxidant pathway in worms (Figure 12).

A growing number of patients with Alzheimer’s disease (AD) are being diagnosed as the population ages worldwide (Association, 2012). Symptoms of AD include memory problems, low lifespan, and impaired cognition, which is closely related with abnormal neuronal death, intracellular amyloid-β (Aβ) deposition and neurofibrillary tangles (Abbas and Wink, 2010; Singh et al., 2020). The abnormal formation and aggregation of Aβ in the central nervous system are major factors of AD development, which can increase oxidative stress (Zhang et al., 2017), and in turn, exceeded ROS (oxidative stress) also enhances the accumulation of Aβ and causes Aβ toxicity (Shukla et al., 2011). As there is currently no effective therapy for AD, it is necessary to search new compounds that prevent the deposition of Aβ by reduction of oxidative stress or activate of disease modifying pathways for reduction the incidence of AD. In this study, the CL4176s model was used to investigate the effects of CXL and CXR on alleviating symptoms of nematode paralysis, and only CXR effectively reduced the Aβ-induced toxicity, which may be related to their rich phenolic acids, phthalides and terpenes. For example, the neuroprotective effects of caffeic acid and chlorogenic acid by alleviating Aβ amyloid accumulation have been demonstrated in the MPTP intoxicated mouse model (Kuang et al., 2009; Habtemariam, 2016; Xu et al., 2016). Moreover, we determined whether CXR treatment could change the accumulation and mRNA expression levels of Aβ in the CL4176s. The results showed that CXR treatment significantly reduced protein accumulation, but did not increase *amy-1* gene expression. Therefore, we propose that a reduction in Aβ aggregation may be the main cause of the improved behavior/phenotype of CL4176s after treatment with CXR. Next, our investigation of the effects of CXR on ROS production and SOD activity revealed that CXR treatment significantly decreased Aβ-induced ROS production and enhanced the activity of SOD in the Aβ transgenic worms (Figures 10A,B). Thus, the Aβ aggregation improvement after CXR treatment may be related to the activation of antioxidant signaling pathways in the nematodes. This hypothesis was tested further here by determining whether some of the relevant regulators were involved in the CXR-mediated reduction of Aβ toxicity. It has been reported that SKN-1, HSF-1, and DAF-16 play key roles in the regulation of Aβ toxicity through activation of antioxidant signaling pathways (Dostal et al., 2010; Dillin and Cohen, 2011; Guo et al., 2016). Moreover, sod-3, hsp-16.1, hsp-70 and hsp-16.2, as their target genes, encode proteins responsible for antioxidant defences to remove excessive ROS and relieve Aβ toxicity (Murphy et al., 2003). Heat shock proteins can repair or remove abnormally folded proteins and are an important element in maintaining intracellular protein homeostasis. For example, HSP-16.1 encodes HSP-16, which directly interacts with Aβ peptide and inhibits oligomerization pathways, reducing toxic species formation in *C. elegans* (Ai et al., 2017). In the CXR group, the expression lever of *hsf-1* and *daf-16* and their downstream antioxidant genes *hsp-16.1*, *hsp-70*, *sod-3*, and *sod-5* was significantly upregulated in the CL4176s, but not skn-1, *hsp-16.2*, and *hsp-16.2* (Figure 10C). Therefore, the findings suggest that CXR neuroprotection is mediated *via* activation hsf-1 and daf-16, as well as their downstream genes (*hsp-16.1*, *hsp-70*, *sod-3*, and *sod-5*). The proposed signaling pathway in CL4176s for CXR function is illustrated in Figure 12.

Toxicity assessment is an essential step for substance to develop be developed into potential functional foods or drugs to alleviate the process of aging or AD development. Our results showed CXL and CXR treatment did not lead acute toxicity in a range of concentrations between 250 and 1,000 μg/ml. In addition, the growth as well as reproduction and fertility performance were not affected at the dose of 0.5 mg/ml, and the treatment also increased the lifespan and the motility of worms. Overall, the CXL and CXR used in this study did not show obvious short-term and long-term toxicity, which is the first investigation providing evidence on the *Ligusticum chuanxiong* leaves and rhizome extracts toxicity.

## 5 Conclusion

In conclusion, the *Ligusticum chuanxiong* leaves (CXL) *and* rhizome (CXR) extracts exhibited antioxidant activity and prolonged the lifespan without obvious side effect in *C. elegans* N2. Moreover, CXR treatment reduced the β-amyloid peptide-induced paralysis phenotype and Aβ aggregation, and alleviated chemosensory behavior dysfunction in neurodegenerative *C. elegans* models. The potential mechanisms of the anti-aging effect of CXs and the neuroprotective effect CXR may be related to the activation of antioxidant pathways, including promoting the overexpression of some antioxidant genes, activating the antioxidant enzyme system, and finally alleviating ROS accumulation. Moreover, considerable phytochemical composition was identified in CX, such as phthalides, alkaloids, organic acids, terpenes and polyphenols, although which substances play an important role in the anti-aging and neuroprotective effects still need more detailed research. Overall, our findings confirmed the antioxidant, anti-aging and neuroprotective properties of *Ligusticum chuanxiong* leaves and rhizome extracts, which provide a scientific basis for expanding the use of *Ligusticum chuanxiong* in pharmaceutical and food industry. However, more *in vivo* interventions with complex model organisms are needed to support the biological effects of *Ligusticum chuanxiong* in the future.

## Data Availability

The original contributions presented in the study are included in the article/Supplementary Material, further inquiries can be directed to the corresponding author.

## Abbreviations

Aβ, β-amyloid peptide; CAT, catalase; CX, Ligusticum chuanxiong Hort.; CXL, Ligusticum chuanxiong leaves extract; CXR, Ligusticum chuanxiong rhizome extract; GSH-Px, glutathione peroxidase; MDA, malondialdehyde; Res, resveratrol; SOD, superoxide dismutase; ROS, reactive oxygen species; VE, vitamin E.
