# Supplementary material for: Ligusticum chuanxiong Hort as a medicinal and edible plant foods: Antioxidant, anti-aging and neuroprotective properties in Caenorhabditis elegans
Source: Front Pharmacol. 2022 Oct 26;13:1049890. doi: 10.3389/fphar.2022.1049890 (PMC9643709; doi:10.3389/fphar.2022.1049890)
Supplement: Supplementary file 1 [file DataSheet1.docx]

**Table. S1** Primer sequences for qRT-PCR analysis

| **Gene** | **Primer** |
| --- | --- |
| *hsp-16.1* | CTGAATCTTCTGAGATTGTTAAC (F)  TTTGTTCAACGGGCGCTTGC (R) |
| *hsp-16.2* | CTGCAGAATCTCTCCATCTGAGTC (F)  AGATTCGAAGCAACTGCACC (R) |
| *hsf-1* | GAAATGTTTTGCCGCATTTT (F) |
|  | CCTTGGGACAGTGGAGTCAT (R) |
| *sod-3* | CCAACCAGCGCTGAAATTCAATGG (F)  GGAACCGAAGTCGCGCTTAATAGT (R) |
| *sod-5* | GAACTGCTGTCTTCGGAACTG (F)  CCATGAAGTCCTGGTGACAAT (R) |
| *hsp-12.6* | TGGCCACTTCAAAAGGGAG (F)  CTCTTTTGGGAGGAAGTTATGG (R) |
| *skn-1* | CACGCCGTCAGCGAAGTA(F)  ATGCTCGGTGAGTATTGG(R) |
| *daf-16* | CTTCAAGCCAATGCCACTACC (F)  GGAGATGAGTTGGATGTTGATAGC (R) |
| *daf-2* | GGATAAAGGCGAATCAAAGTGTC (F) |
|  | CGATACACTTTCCCTTGTGATAGAC (R) |
| *hsp-70* | GAAGGGACGACTCTCTCAAGCT (F)  CGATCTCGTTGTGCTGCG (R) |
| *amy-1* | CCGACATGACTCAGGATATGAAGT (F)  CACCATGAGTCCAATGATTGCA (R) |
| *act-1* | TCCAAGAGAGGTATCCTTAC (F)  CGGTTAGCCTTTGGATTGAG (R) |

**Table S2** Identified compounds from CXL and CXR by UPLC-ESI-QTOF-MS analyses in positive and negative ionization modes

| NO | Proposed compounds | Proposed Formula | Molecular Weight | [M-H]^−/+^ (M/Z) | Rt (min) | MS/MS fragments | CXR | CXLL |
| --- | --- | --- | --- | --- | --- | --- | --- | --- |
| Phthalides | | | | | | | | |
| 1 | Ligustilide | C_12_H_14_O_2_ | 190.09 | 191.10 [M-H]^+^ | 8.47 | 173、145、117 | * |  |
| 2 | Senkyunolide A | C_12_H_16_O_2_ | 192.11 | 193.12 [M-H]^+^ | 4.60 | 147、91、105、119 | * | * |
| 3 | Levistilide A | C_24_H_28_O_4_ | 380.20 | 381.20 [M-H]^+^ | 11.36 | 191 | * |  |
| 4 | Sedanolide | C_12_H_18_O_2_ | 194.13 | 195.13 [M-H]^+^ | 9.55 | 79、149、125 | * | * |
| Alkaloids | | | | | | | | |
| 5 | Thalsimine | C_38_H_40_N_2_O_7_ | 636.28 | 637.29 [M-H]^+^ | 4.66 | 154、473、316 |  | * |
| 6 | Lycorine-monoacetate | C_18_H_19_NO_5_ | 329.13 | 330.14 [M-H]^+^ | 4.66 | 266、107 | * |  |
| 7 | Crotonoside | C_10_H_13_N_5_O_5_ | 283.09 | 284.10 [M-H^]+^ | 1.33 | 152、268 | * | * |
| 8 | Dictamnine | C_12_H_9_NO_2_ | 199.06 | 200.07 [M-H]^+^ | 6.04 | 172 | * |  |
| 9 | Pyrrole-2-carboxylic acid | C_5_H_5_NO_2_ | 111.03 | 112.04 [M-H]^+^ | 1.17 | 69、95 | * | * |
| 10 | Choline | C_5_H_13_NO | 103.10 | 104.10 [M-H]^+^ | 10.17 | 60、79 | * | * |
| 11 | Uracil | C_4_H_4_N_2_O_2_ | 112.03 | 113.03 [M-H]^+^ | 1.36 | 70、96 | * | * |
| 12 | Adenine | C_5_H_5_N_5_ | 135.05 | 134.05 [M-H]^-^ | 1.31 | 107 | * |  |
| 13 | Adenosine | C_10_H_13_N_5_O_4_ | 267.10 | 266.09 [M-H]^-^ | 1.31 | 134 | * |  |
| 14 | Scopoletin | C_10_H_8_O_4_ | 192.04 | 191.03 [M-H]^-^ | 4.93 | 176 | * |  |
| 15 | Salsolinol | C_10_H_13_NO_2_ | 179.09 | 180.10 [M-H]^+^ | 8.35 | 137、179、91 |  | * |
| 16 | Nicotinic acid | C_6_H_5_NO_2_ | 123.03 | 124.04 [M-H]^+^ | 0.95 | 80、95 | * | * |
| 17 | Crotonoside | C_10_H_13_N_5_O_5_ | 283.09 | 284.10 [M-H]^+^ | 1.33 | 152、268 | * | * |
| Organic acids and its esters: fatty acids (18-23) , organic acid esters(24-26) and Organic acids(27-39) | | | | | | | | |
| 18 | Palmitic acid | C_16_H_32_O_2_ | 256.24 | 255.23 [M-H]^-^ | 13.42 | 166、94、192 | * | * |
| 19 | Oleic Acid | C_18_H_34_O_2_ | 282.26 | 281.25 [M-H]^-^ | 13.58 | 199、191、95 |  | * |
| 20 | Linoleic Acid | C_18_H_32_O_2_ | 280.24 | 279.23 [M-H]^-^ | 12.88 | 166、108、191 | * | * |
| 21 | Elaidic acid | C_18_H_34_O_2_ | 282.26 | 283.26 [M-H]^+^ | 10.76 | 191、166 | * | * |
| 22 | 8,11,14-Eicosatrienoic acid | C_20_H_34_O_2_ | 306.26 | 307.26 [M-H]^+^ | 10.33 | 81、67、95 | * | * |
| 23 | Crotonic acid | C_4_H_6_O_2_ | 86.04 | 87.04 [M-H]^+^ | 0.79 | 69 | * | * |
| 24 | Methyl gallate | C_8_H_8_O_5_ | 184.04 | 183.03 [M-H]^-^ | 2.56 | 83、111、138 | * | * |
| 25 | Ethyl caffeate | C_11_H_12_O_4_ | 208.07 | 207.07 [M-H]^-^ | 6.40 | 135 | * | * |
| 26 | Ethyl ferulate | C_12_H_14_O_4_ | 222.09 | 221.08 [M-H]^-^ | 4.33 | 206、191 |  | * |
| 27 | ascorbic acid | C_6_H_8_O_6_ | 176.03 | 175.02 [M-H]^+^ | 0.83 | 146、118 |  | * |
| 28 | Cinnamic acid | C_9_H_8_O_2_ | 148.05 | 149.06 [M-H]^+^ | 8.93 | 121 | * | * |
| 29 | Benzoic acid | C_7_H_6_O_2_ | 122.04 | 123.04 [M-H]^+^ | 8.80 | 79、105 | * | * |
| 30 | Tiglic acid | C_5_H_8_O_2_ | 100.05 | 101.06 [M-H^]+^ | 0.86 | 84、73、56 | * | * |
| 31 | Citric acid | C_6_H_8_O_7_ | 192.03 | 191.02 [M-H]^-^ | 0.87 | 85 | * | * |
| 32 | D-(-)-Quinic acid | C_7_H_12_O_6_ | 192.06 | 191.06 [M-H]^-^ | 3.59 | 85 | * |  |
| 33 | DL-Malic acid | C_4_H_6_O_5_ | 134.02 | 133.01 [M-H]^-^ | 1.00 | 115、71、 | * | * |
| 34 | 2-Hydroxy-4-(methylthio)butyric acid | C_5_H_10_O_3_S | 150.03 | 149.03 [M-H]^-^ | 3.26 | 147、121、77 |  | * |
| 35 | 2-Isopropylmalic acid | C_7_H_12_O_5_ | 176.07 | 175.06 [M-H]^-^ | 3.63 | 115、85、83 | * |  |
| 36 | 6-Hydroxycaproic acid | C_6_H_12_O_3_ | 132.08 | 131.07 [M-H]^-^ | 4.33 | 85 |  | * |
| 37 | Salicylic acid | C_7_H_6_O_3_ | 138.03 | 137.02 [M-H]^-^ | 5.58 | 108、93 | * | * |
| 38 | Sebacic acid | C_10_H_18_O_4_ | 202.12 | 201.11 [M-H]^-^ | 5.98 | 139、201、183、154 |  | * |
| 39 | Vanillin | C_8_H_8_O_3_ | 152.05 | 151.04 [M-H]^+^ | 4.75 | 105、69、79 |  | * |
| Terpenes | | | | | | | | |
| 40 | α-Methylene-γ-butyrolactone | C_5_H_6_O_2_ | 98.04 | 99.04 [M-H]^+^ | 0.81 | 78、69、62 | * | * |
| 41 | Guaiazulene | C_15_H_18_ | 198.14 | 199.15 [M-H]^+^ | 10.03 | 128 |  | * |
| 42 | Albiflorin | C_23_H_28_O_11_ | 480.16 | 481.17 [M-H]^-^ | 5.29 | 437、395 | * |  |
| 43 | Catalpol | C_15_H_22_O_10_ | 362.12 | 361.11 [M-H]^-^ | 1.85 | 89、179、135、71、119 | * |  |
| Polyphenols: phenolic acids (44-57), Flavonoids (58-66), Anthocyanidins (67) and phenols (68-78) | | | | | | | | |
| 44 | Ferulic acid | C_10_H_10_O_4_ | 194.06 | 195.07 [M-H]^+^ | 3.43 | 177、145 | * | * |
| 45 | Caffeic acid | C_9_H_8_O_4_ | 180.04 | 181.05 [M-H]^+^ | 3.58 | 177、145 | * | * |
| 46 | Chlorogenic acid | C_16_H_18_O_9_ | 354.10 | 353.09 [M-H]^+^ | 3.59 | 191 | * | * |
| 47 | Sinapinic acid | C_11_H_12_O_5_ | 224.07 | 225.08 [M-H]^+^ | 3.95 | 56、123 | * | * |
| 48 | Isovanillic acid | C_8_H_8_O_4_ | 168.04 | 169.05 [M-H]^+^ | 3.30 | 111、93、65 | * | * |
| 49 | Gallic acid | C_7_H_6_O_5_ | 170.02 | 169.01 [M-H]^-^ | 1.79 | 125 |  | * |
| 50 | Gentisic acid | C_7_H_6_O_4_ | 154.03 | 155.03 [M-H]^+^ | 2.01 | 137、111、81 | * | * |
| 51 | Xanthurenic acid | C_10_H_7_NO_4_ | 205.04 | 204.03 [M-H]^-^ | 2.64 | 102、71 |  | * |
| 52 | 5-Hydroxyindole-3-acetic acid | C_10_H_9_NO_3_ | 191.06 | 190.05 [M-H]^-^ | 4.38 | 120、164、111 |  | * |
|  |  |  |  |  |  |  |  |  |
| 53 | 2-Hydroxycinnamic acid | C_9_H_8_O_3_ | 164.05 | 163.04 [M-H]^-^ | 7.95 | 119、91 | * | * |
| 54 | 3-(4-Hydroxyphenyl)lactate | C_9_H_10_O_4_ | 182.06 | 181.05 [M-H]^-^ | 3.34 | 137、108、93 |  | * |
| 55 | 3,4-Dihydroxyphenylacetic acid | C_8_H_8_O_4_ | 168.04 | 167.03 [M-H]^-^ | 3.92 | 123、95 | * |  |
| 56 | 3-Dimethylallyl-4-hydroxybenzoic acid | C_12_H_14_O_3_ | 206.09 | 205.09 [M-H]^-^ | 7.70 | 161、138、106 | * | * |
| 57 | 3-Hydroxyphenylacetic acid | C_8_H_8_O_3_ | 152.05 | 151.04 [M-H]^-^ | 3.41 | 161、106、138 | * | - |
| 58 | Formononetin | C_16_H_12_O_4_ | 268.07 | 269.08 [M-H]^+^ | 4.74 | 254 | * | * |
| 59 | (-)-Epicatechin | C_15_H_14_O_6_ | 290.08 | 289.07 [M-H]^-^ | 7.14 | 221、 177 | * | * |
| 60 | 4-Hydroxy-2',3,4',6'-tetramethoxychalcone | C_19_H_20_O_6_ | 344.13 | 343.12 [M-H]^-^ | 4.47 | 257 | * | * |
| 61 | Genistein | C_15_H_10_O_5_ | 270.05 | 269.05 [M-H]^-^ | 5.24 | 123、179、169、191、255 |  | * |
| 62 | Loureirin B | C_18_H_20_O_5_ | 316.13 | 315.12 [M-H]^-^ | 5.67 | 268、158、71 |  | * |
| 63 | Methyl hesperidin | C_29_H_36_O_15_ | 624.21 | 623.20 [M-H]^-^ | 4.16 | 327、165、149、159 | * | * |
| 64 | Loureirin A | C_17_H_18_O_4_ | 286.12 | 285.11 [M-H]^-^ | 6.49 | 291、133、93 |  | * |
| 66 | Flavokawain A | C_18_H_18_O_5_ | 314.12 | 313.11 [M-H]^-^ | 5.79 | 223、251、159、119 | * | * |
| 67 | Procyanidin B2 | C_30_H_26_O_12_ | 578.14 | 577.14 [M-H]^-^ | 6.30 | 193、178、143、134 | * | * |
| 68 | Purpureaside C | C_35_H_46_O_20_ | 786.26 | 785.25 [M-H]^-^ | 3.83 | 165、579 |  | * |
| 69 | 2-Amino-1,3,4-octadecanetriol | C_18_H_39_NO_3_ | 317.29 | 318.30 [M-H]^+^ | 7.77 | 256、88、102 |  | * |
| 70 | 2-Aminopheno | C_6_H_7_NO | 109.05 | 110.06 [M-H]^+^ | 7.63 | 67、82 |  | * |
| 71 | hydroquinone | C_6_H_6_O_2_ | 110.04 | 111.04 [M-H]^+^ | 8.80 | 67、55 | * | * |
| 72 | 4-Hydroxybenzoic acid | C_7_H_6_O_3_ | 138.03 | 139.04 [M-H]^+^ | 3.47 | 121、95 | * | * |
| 73 | 1-Naphthol | C_10_H_8_O | 144.06 | 145.06 [M-H]^+^ | 7.49 | 117、97、103 | * | * |
| 74 | Daphnetin | C_9_H_6_O_4_ | 178.03 | 179.03 [M-H]^+^ | 3.68 | 119、91、147、161 | * |  |
| 75 | Fraxetin | C_10_H_8_O_5_ | 208.04 | 209.04 [M-H^]+^ | 4.08 | 194、149、107 | * | * |
| 76 | 4-Vinylphenol | C_8_H_8_O | 120.06 | 119.05 [M-H]^-^ | 4.79 | 118、93 | * | * |
| 77 | 4-Hydroxybenzaldehyde | C_7_H_6_O_2_ | 122.04 | 121.03 [M-H]^-^ | 4.42 | 108、93 |  | * |
| 78 | Scopoletin | C_10_H_8_O_4_ | 192.04 | 191.03 [M-H]^-^ | 4.93 | 176、148、104 |  | * |
| Others: aldehydes、Ketones、Sterols (79-88), amino acids (89-91) and monose (92-98) | | | | | | | | |
| 79 | 7-Hydroxycoumarine | C_9_H_6_O_3_ | 162.03 | 163.04 [M-H]^+^ | 3.15 | 135、117、89 | * | * |
| 80 | 2,8-Quinolinediol | C_9_H_7_NO_2_ | 161.05 | 162.05 [M-H]^+^ | 4.21 | 135、145、117、89 | * | * |
| 81 | Apocynin | C_9_H_10_O_3_ | 166.06 | 167.07 [M-H]^+^ | 4.67 | 135、104、129 | * | * |
| 82 | N-Benzyl-N-hydroxy-1-phenylmethanamine | C_14_H_15_NO | 213.12 | 214.12  [M-H]^+^ | 5.93 | 186、168 | * |  |
| 83 | Carveol | C_10_H_16_O | 152.12 | 153.13 [M-H]^+^ | 5.03 | 79、97、107 | * | * |
| 84 | Benzyl Alcohol | C_7_H_8_O | 108.06 | 109.07 [M-H^]+^ | 5.65 | 67、81、91 | * | * |
| 85 | 3-Pyridinemthanol | C_6_H_7_NO | 109.05 | 110.06 [M-H]^+^ | 0.85 | 83、93 | * | * |
| 86 | trans-Cinnamaldehyde | C_9_H_8_O | 132.06 | 133.06 [M-H]^+^ | 6.73 | 105、91、118 | * | * |
| 87 | Anethole | C_10_H_12_O | 148.09 | 147.08 [M-H]^-^ | 6.99 | 147、61、85、116、102 | * | - |
| 88 | Carvone | C_10_H_14_O | 150.10 | 151.11 [M-H]^+^ | 6.92 | 81、96 | * | * |
| 89 | Methionine | C_5_H_11_NO_2_S | 149.05 | 150.06 [M-H]^+^ | 0.87 | 149、104、56、121、133 | * | * |
| 90 | L-Histidine | C_6_H_9_N_3_O_2_ | 155.07 | 156.08 [M-H^]+^ | 2.15 | 110、95、83 | * |  |
| 91 | L-Tyrosine | C_9_H_11_NO_3_ | 181.07 | 180.07 [M-H]^-^ | 1.22 | 163、119、72、93 | * | * |
| 92 | Arabinose | C_5_H_10_O_5_ | 150.05 | 149.04 [M-H]^-^ | 5.31 | 59、121、71、89 | * | * |
| 93 | D-(-)-Arabinose | C_5_H_10_O_5_ | 196.06 | 195.05 | 0.81 | 75、129、87 | * | * |
| 94 | D-(-)-Fructose | C_6_H_12_O_6_ | 180.06 | 179.06 [M-H]^-^ | 0.77 | 134、91、122、150 | * | * |
| 95 | D-(+)-Maltose | C_12_H_22_O_11_ | 342.12 | 341.11 [M-H]^-^ | 0.81 | 89、71、101、119、179 | * | * |
| 96 | D-Raffinose | C_18_H_32_O_16_ | 504.17 | 503.16 [M-H]^-^ | 1.08 | 113、89、71 | * |  |
| 97 | Sucrose | C_12_H_22_O_11_ | 342.12 | 341.11 [M-H]^-^ | 3.88 | 179、135、89、71 | * | - |
| 98 | L(-)-Fucose | C_6_H_12_O_5_ | 164.07 | 163.06 [M-H]^-^ | 0.85 | 119、91、139 | * | * |

*：Signal detected

**Table. S3** Effects of CXs on the viability of wild-type *C. elegans*: The results are presented as mean of % viability ±SEM. Not significance differences between treatment and control groups found (p>0.05)

| Extract | Concentration（µg/mL） | Survival rate（%） | *p*-Value |
| --- | --- | --- | --- |
|  | Control | 92±2 |  |
| CXL | 250 | 94±1 | >0.05 |
|  | 500 | 96±4 | >0.05 |
|  | 750 | 94±1 | >0.05 |
|  | 1000 | 94±2 | >0.05 |
| CXR | 250 | 95 | >0.05 |
|  | 500 | 94±1 | >0.05 |
|  | 750 | 95±4 | >0.05 |
|  | 1000 | 94±1 | >0.05 |

| Stressors | Group | Mean time^(1)^ | Median time^(2)^ | Median time^(3)^ | P-Value^(4)^ |
| --- | --- | --- | --- | --- | --- |
| N2(H_2_O_2_) | CK | 2.53±0.10_b_ | 2.50±0.00_a_ | 5.33±0.58_a_ |  |
|  | CXL | 3.12±0.22_a_ | 2.50±0.00_a_ | 6.00±1.00_a_ | <0.001 |
|  | CXR | 3.33±0.19_a_ | 2.83±0.58_a_ | 6.00±1.00_a_ | <0.001 |
|  | Res | 3.47±0.4_a_ | 3.17±0.58_a_ | 7.34±1.15_a_ | <0.001 |
| N2(paraquat) | CK | 2.17±0.54_b_ | 2.16±0.58_a_ | 5.33±0.58_a_ |  |
|  | CXL | 2.06±0.35_b_ | 2.16±0.58_a_ | 6.00±1.00_a_ | 0.24 |
|  | CXR | 3.09±0.04_a_ | 2.67±0.76_a_ | 6.00±1.00_a_ | <0.001 |
|  | Res | 2.63±0.26_ab_ | 2.5±0.87_a_ | 6.67±0.58_a_ | <0.01 |
| N2(37℃） | CK | 5.83±0.64_c_ | 5.67±1.04_a_ | 12.30±0.28_a_ |  |
|  | CXL | 6.59±0.35_bc_ | 6.50±1.00_a_ | 14.00±1.00_a_ | <0.05 |
|  | CXR | 6.96±0.38_ab_ | 6.83±0.29_a_ | 14.00±1.00_a_ | <0.01 |
|  | Res | 7.48±0.16_a_ | 7.17±0.29_a_ | 15_a_ | <0.001 |
| N2 | CK | 17.71±1.34_c_ | 17.17±1.70_a_ | 24.67±1.70_b_ |  |
|  | CXL | 18.74±3.07_ab_ | 18.17±3.77_a_ | 28.00±2.16_a_ | <0.001 |
|  | CXR | 19.21±1.63_a_ | 18.50±2.83_a_ | 28.67±1.70_a_ | <0.001 |
|  | Res | 18.2±0.11_b_ | 17.67±2.52_a_ | 29.67±1.15_a_ | <0.01 |

**Table. S4** Statistical analysis of the lifespan of *C. elegans* under pressure

All data are presented as the mean ± SD, and different letters in columns indicate that the values are significantly different (р<0.05).

(1) Mean survival time: MLS =1/n∑_j_ (Xj+X_j+1_)/2d_j_, where j is the age category, d_j_ is the number of worms that died in the age interval (x_j_, x_j+1_), and n is the total number of worms.

(2) The median lifespan is the time at which fraction survival equals 50%.

(3) The maximum lifespan is the time at which fraction survival equals 0%.

(4) P-value was calculated using the log-rank test by comparing the CXL or CXR-treated group with control.

| Treatment | Mean  paralysis | Median  paralysis(PT_50_) | Maximum  Paralysis | p-Value |
| --- | --- | --- | --- | --- |
| CK | 32.02±0.61^b^ | 31.33±0.58^b^ | 38^b^ |  |
| CXL | 32.90±0.62^ab^ | 32.00±1.00^ab^ | 39.33±1.15^ab^ | >0.05 |
| CXR | 33.19±0.73^ab^ | 33.67±1.54^a^ | 39.33±1.15^ab^ | <0.01 |
| Res | 34.09±0.53^a^ | 33.67±1.54^a^ | 40^a^ | <0.001 |

**Table S5** Statistical analysis of paralysis of *C. elegans* CL4176
